# Neural Basis of Etiopathogenesis and Treatment of Cervicogenic Orofacial Pain

**DOI:** 10.3390/medicina58101324

**Published:** 2022-09-21

**Authors:** Jiří Šedý, Mariano Rocabado, Leonardo Enrique Olate, Marek Vlna, Radovan Žižka

**Affiliations:** 1 3DK Clinic, U Zdravotniho Ustavu 2213/8, 10000 Prague, Czech Republic; 2 Institute of Dentistry and Oral Sciences, Faculty of Medicine, Palacký University, Palackého 12, 77200 Olomouc, Czech Republic; 3 Institute of Anatomy, Second Faculty of Medicine, Charles University, V Uvalu 84, 15006 Prague, Czech Republic; 4 Facultad de Ciencias de la Rehabilitación, Universidad Andres Bello, Repůblica 239, Santiago 8370035, Chile; 5 Rocabado Institute, Camino El Alba 8760, Las Condes, Región Metropolitana de Santiago, Santiago 87608760, Chile

**Keywords:** cervicogenic, pain, temporomandibular joint, posture, physiotherapy

## Abstract

(1) *Background and Objectives*: The aim of this narrative review was to analyze the neuroanatomical and neurophysiological basis of cervicogenic pain in cervico-cranial pain syndromes, focusing particularly on cervico-orofacial syndromes as a background for the proper diagnosis and non-surgical treatment. Relevant literature on the topic from past 120 years has been surveyed. (2) *Material and Methods*: We surveyed all original papers, reviews, or short communications published in the English, Spanish, Czech or Slovak languages from 1900 to 2020 in major journals. (3) *Results*: The cervicogenic headache originates from the spinal trigeminal nucleus where axons from the C_1_–C_3_ cervical spinal nerves and three branches of the trigeminal nerve converge (trigeminocervical convergence) at the interneurons that mediate cranio-cervical nociceptive interactions. The role of the temporomandibular joint in the broad clinical picture is also important. Despite abundant available experimental and clinical data, cervicogenic orofacial pain may be challenging to diagnose and treat. Crucial non-surgical therapeutic approach is the orthopedic manual therapy focused on correction of body posture, proper alignment of cervical vertebra and restoration of normal function of temporomandibular joint and occlusion. In addition, two novel concepts for the functional synthesis of cervico-cranial interactions are the tricentric concept of mouth sensorimotor control and the concept of a cervicogenic origin of bruxism. (4) *Conclusions*: Understanding the basis of neuroanatomical and neurophysiological neuromuscular relations enables an effective therapeutic approach based principally on orthopedic manual and dental occlusal treatment.

## 1. Introduction

Cervicogenic headache, or more broadly, cervicogenic orofacial pain (COP), represents an important clinical entity defined as a secondary, lateralized non-throbbing headache caused by nociceptive sources in the cervical spine [1]. Its negative impact on quality of life is substantial and may be comparable to those of migraines and episodic tension-type headaches [2,3]. Cervicogenic orofacial syndrome is characterized by pain that begins in the neck or occipital region and progresses to adjacent regions of the face and head [4,5]. The anatomical basis for this clinical pattern likely relates to a convergence of the upper cervical and trigeminal nociceptive afferents within the trigeminocervical neural complex [6]. 

Regarding basic neuroscience, COP is probably one of the best-understood categories of common headaches. Its etiopathogenetic mechanisms are largely known, based mainly on the convergence on afferent nerve fibers from first three cervical nerves and trigeminal branches on the interneurons of the spinal trigeminal nucleus, as a source of referred pain from cervical region to trigeminal area [4,5,6]. The pain can be induced experimentally in healthy volunteers. It is diagnostically helpful that in some patients, COP can be temporarily relieved by blocking the cervical joints or nerves with local anesthetics [5].

The Cervicogenic Headache International Study Group formally identified the clinical syndrome as a separate entity in 1998. Consequently, it was included in the second and third edition of the International Headache Society classification of Headache in 2004 [7,8] (Table 1). The Study Group classified cervicogenic headache as a side-locked orofacial pain (OP) worsened by diverse stimuli such as neck movements, sustained improper neck positioning, restricted range of cervical spine motion, or ipsilateral shoulder and arm pain [9]. Despite this relatively strict definition, COP may be challenging to diagnose clinically. For example, a sharp pain in the occipital region may reflect neuralgia of the occipital nerve that can mimic COP [10]. Signs and symptoms associated with migraine headaches or other symptoms of vascular headaches, including neck pain, nausea, vomiting, photophobia, and phonophobia can imitate COP [9,11,12]. Despite abundantly available experimental and clinical data, therapeutic modalities based on specific etiopathogenic mechanisms of COP have not been comprehensively elucidated and are the subject of this review. Further text is based elaborating the non-surgical treatment modalities, mainly based on orthopedic manual therapy, based on the author’s own concepts, such as Rocabado’s tricentric concept of mouth sensorimotor control, the importance of posture, proper alignment of cervical vertebrae and the role of concomitant conservative temporomandibular joint therapy. Moreover, the author’s own concept of bruxism etiopathogenesis pathway is presented as well.

## 2. Data Collection and Analysis

We surveyed all original papers, reviews, or short communications published in the English, Spanish, Czech or Slovak languages from 1900 to 2020 in major journals. Studies were included when they dealt with COP experimentally or clinically based on Medline, Web of Science, OVID, and Google Scholar searches. The parameters studied were mainly neuroanatomy, neurophysiology, etiology, pathogenesis, clinical appearance, and therapeutic modalities. We particularly focused on COP syndromes with more emphasis on cervico-orofacial syndromes.

## 3. Epidemiology

The prevalence of COP is between 1–4.1% in the general population but could be as high as 17.5% among patients with severe headaches [9,12,13,14]. The highest prevalence is in the patients with whiplash injury where it could be as high as 53% [15].

## 4. Clinical Anatomy of Cervico-Cranial Junction

The cervico-cranial complex represents a crucial part of the axial (postural) system. It is involved in many functions including the primary movements of the head, and participating in remote secondary functions such as positioning of the head in accordance with visual and vestibulocochlear inputs, movements of the mandible at the temporomandibular joint, pharyngeal and laryngeal functions, and others. Clinically, the cranio-mandibulo-cervical complex includes the first three vertebrae, skull, mandible, and hyoid bone, together with the muscles, ligaments, fasciae, and other structures involved in their movement [16].

Among the first three cervical vertebrae, only the third represents a typical cervical vertebra with a distinct body, vertebral arch, and typical processes. The C_1_ vertebra (*atlas*) lacks a typical vertebral body, being composed of anterior and posterior arches only together with paired lateral masses. The C_2_ vertebra (*axis*) serves as an axis for rotation of the *atlas* and head around its strong odontoid process (*dens*), projecting cranially from the superior surface of the body. It is retained in position by the strong transverse ligament of the *atlas*. Further, the tip of the *atlas* is connected to the occipital bone with paired alar ligaments, approximately 11 mm long, and thick collagenous cords. The *atlas* is connected to the occipital bone by two atlanto-occipital joints, developed between occipital condyles and articular surfaces on the superior aspects of the lateral masses of the *atlas*. In this joint, the main movements are flexion and extension (range 16–21°), with limited lateral flexion (range 3°) and rotation (range 6°) [17]. The connection of the *atlas* and *axis* is in the abovementioned, unpaired, median atlanto-axial joint between the *dens axis* and posterior surface of the anterior arch of the *atlas* and paired lateral atlanto-axial zygapophyseal joints. The main movement of this joint is rotation with a normal range of approximately 41° [17]. Atlanto-axial rotation is limited by clinically very important alar ligaments, the left becoming taut on rotation to the right and vice versa. The slightly upward movement of the C_2_ vertebra during rotation facilitates a wide range of movement by reducing the tension in the alar ligaments, as it also does in the capsules and accessory ligaments of the lateral atlanto-axial joints [18]. Between the second and third cervical vertebrae, typical intervertebral disc together with paired, lateral, zygapophyseal joints are developed. Together with the four other cervical vertebrae, these structures form a typical convexity directed forward, called cervical lordosis. Moreover, the first two thoracic vertebrae are involved; thus, typical physiological cervical lordosis extends from the *atlas* to the Th_2_ vertebra, with its maximum bending being between the C_4_ and C_5_ vertebrae.

### 4.1. Spinal Nerves

The cervical vertebrae form the cranial part of the vertebral canal, which encloses the spinal cord giving rise to the spinal nerves, which form by a fusion of motor ventral and sensory dorsal spinal roots to pass as one nerve bundle through the intervertebral foramina. The first cervical spinal nerve (C_1_) passes between the occipital bone and C_1_ vertebra. The last cervical spinal nerve (C_8_) passes between the last cervical vertebra and the first thoracic vertebra—the reason why humans have eight cervical spinal nerves but only seven cervical vertebrae. After passing through the intervertebral foramen (or corresponding space) the spinal nerves emerge laterally and give rise to anterior and posterior nerve branches (*rami*). Importantly, the peripheral branches of the cervical spinal nerves supply neck muscles, which are functionally related to movements of the cervical spine, head, and mandible.

### 4.2. Spinal System and Trigeminocervical Convergence

The trigeminal nerve is the thickest cranial nerve, and it contains approximately 180,000 nerve fibers. It is a very huge somatosensory part (*radix sensoria*, *portio major*) that innervates the skin of the face, all teeth, oral, nasal, and paranasal mucosa, anterior two-thirds of the tongue, orbit, part of nasopharynx, mucosa of the Eustachian tube, temporomandibular joint (TMJ), majority of the dura mater, lateral part of the tympanic membrane, external auditory meatus, and small part of the auricle [18,19,20]. The most intense pain stimuli in this large innervating area are provided by free nerve endings from the dental pulp and cornea. The borders of the innervating areas of particular branches (ophthalmic, maxillary, and mandibular) are *rima palpebrarum* and *rima oris*. Posteriorly, the margin remains the line vertex—external auditory meatus—chin. The importance of the trigeminal sensory innervation is very high—approximately 50% of the cortical representation of all sensory inputs from the body comes from the trigeminal system [21]. Trigeminal primary afferents are dendrites of pseudounipolar neurons in trigeminal (semilunar, Gasserian) ganglion and mesencephalic nucleus [22]. The motor part (*radix motoria*, *portio minor*) innervates all chewing muscles (masseter, temporalis, and medial and lateral pterygoids) as well as mylohyoid, anterior belly of digastric, tensor tympani, and tensor veli palatini. In the periphery, the trigeminal nerve system is entered by sympathetic, parasympathetic, and gustatory fibers from other origins.

The trigeminal nerve has three sensory nuclei (trigeminal sensory complex) and one motor nucleus.

#### 4.2.1. Trigeminocervical Nucleus

**Figure 1:**
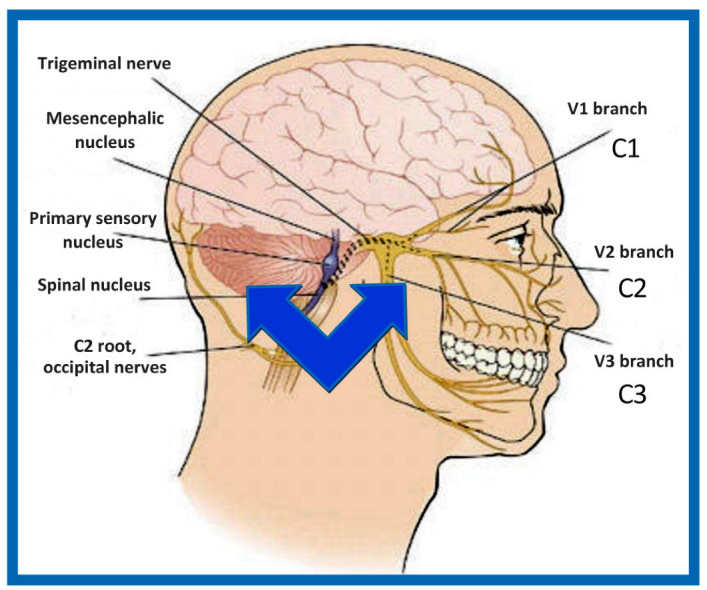
Trigeminocervical convergence. Source: Rocabado Institute, Chile. The convergence of cervical and trigeminal afferents on second-order neurons in the trigeminocervical nucleus may refer to pain from the upper cervical spine into the head and face. Furthermore, bi-directional interactions between the trigeminal and upper cervical afferents may also explain the cervical symptoms of trigeminal origin.

Trigeminocervical nucleus (*nucleus spinalis nervi trigemini*) is an elongated structure, located from the bottom of the pons to upper segments of cervical spinal cord. It has three main parts: (1a) **Subnucleus oralis** (*pars rostralis*) contains afferent fibers from facial region close to the midline, mainly from the oral cavity and nose. (1b) **Subnucleus interpolaris**—contains afferent fibers from the ventrolateral parts of the face, mainly from the cheeks and orbit. (1c) **Subnucleus caudalis**—contains afferent fibers from the lateral parts of the face. Lateral to *subnucleus caudalis*, a bundle of fibers called *tractus spinalis nervi trigemini* is located. This bundle is entered by A-delta and C-type trigeminal fibers leading to pain and thermal stimuli. These fibers are somatotopically organized—most anteriorly, are fibers from the ophthalmic nerve, in the middle, are fibers from the maxillary nerve and dorsally, are fibers from the mandibular nerve. Step by step, these fibers turn medially and enter the trigeminocervical nucleus. To understand COP, it is extremely important to know that the axons of the trigeminocervical nucleus are densely connected with axons of spinal ganglia of C_1_–C_3_ spinal nerves, via interneurons of the trigeminocervical nucleus [5,23]. This pattern is known as **trigeminocervical functional convergence** [24] (Figure 1). Moreover, the referral of sensations is bi-directional [25]. Thus, there is a possibility of referred pain transfer between the C_1_–C_3_ and trigeminal innervation areas [4,5]. Pain originating from the teeth, jaws, or TMJ could thus project into the cervical area, and vice versa. Pain from the C_1_–C_3_ innervation area (e.g., due to improper posture, C_1_/C_2_ vertebra rotation, cervical trauma) could project to the orofacial region, mainly to the forehead, orbit, cheek, TMJ, and ear, causing problems in differential diagnosis with a high risk of misdiagnosis [5,26]. This relation represents the fundamental neuroanatomical basis of COP [5]. Importantly, no relation has been observed between the trigeminal nerve and spinal nerve C_4_ or nerves at lower segments [5]. 

Posterior to the trigeminocervical nucleus, a fine bundle of somatosensory afferent fibers from facial, glossopharyngeal, and vagus cranial nerves are located, known as the **spinal nerve tract** (*tractus spinalis nervi trigemini)*. These fibers also end in the trigeminocervical nucleus—this connection has been confirmed both experimentally and clinically [26,27,28]. This connection is highly clinically relevant, because it explains the pathogenic background of referred pain between the orofacial area innervated by the facial, glossopharyngeal, and vagus cranial nerves on one side and the spinal C_1_–C_3_ innervation area on the other side [26]. The knowledge of the organization of nerve fibers in the spinal nerve tract is important for surgical therapy of trigeminal neuralgia—trigeminal tractotomy, employing the destruction of superficially located nerve fibers (for review, see Cetas et al., 2008 [29]). Efferent fibers from the trigeminocervical nucleus run as a ventral trigeminothalamic tract (*tractus trigeminothalamicus ventralis*), together with fibers from the ventrolateral part of the pontine trigeminal nucleus (see below) to the thalamus, from which it runs to the primary sensory cortical area.

#### 4.2.2. Pontine Trigeminal Nucleus

Pontine trigeminal nucleus (*nucleus pontinus*), also called **principal trigeminal nucleus** (*nucleus principalis nervi trigemini*) is the main somatosensory trigeminal nucleus, located in pons Varoli. Strong A-alpha and A-beta myelinated fibers, leading signals from low-threshold mechanoreceptors of the scalp, end in this nucleus. It has two main parts: (1) The **Dorsomedial part** receives signals from the oral cavity and forms the posterior trigeminothalamic tract (*tractus trigeminothalamicus dorsalis*), which leads ipsilaterally into the thalamus. (2) The **Ventrolateral part** receives signals from all areas innervated by the trigeminal nerve and after crossing the midline, it forms (together with axons of the trigeminocervical nucleus—see above) the ventral trigeminothalamic tract (*tractus trigeminothalamicus ventralis*). This nucleus is functionally related to the gracilis and cuneate nuclei and serves to analyze touch, discrimination, and vibration sensations. It also contains small amounts of proprioceptive fibers, mainly from oculomotor muscles [22]. Efferent fibers from the pontine trigeminal nucleus form a distinct bundle, trigeminal lemniscus (*lemniscus trigeminalis*), which runs into the thalamus and from here to primary sensory cortical areas.

#### 4.2.3. Mesencephalic Nucleus

Mesencephalic nucleus (*nucleus mesencephalicus nervi trigemini*) is an elongated mass of gray matter, located close to the *substantia grisea centralis*. It is unique among central nervous system (CNS) nuclei because it is formed by pseudounipolar neurons [18,22]. Thus, it is considered to be the only intraneuraxial ganglion in the human body [30]. It was originally postulated that these large, glutamatergic pseudounipolar neurons entered the *mesencephalon* from the neural crest during the development; however, recent findings have revealed that they originate primarily from the same material as the future *mesencephalon* [31]. The mesencephalic nucleus is, thereby, the only centrally located nucleus comprising first-order neurons, i.e., they receive impulses directly from receptors, without interpolation. Summarily, 80–90% of dendrites receive proprioceptive signals from the muscle spindles (stretch receptors) of the muscles of mastication, and 10–20% of dendrites receive signals from mechanoreceptors of periodontal tissues, mainly from those at the region of the root apex [32]. Although neurons leading signaling from the masticatory muscles are spread diffusely within the nucleus, i.e., without evident somatotopic organization, neurons registering signals from the periodontal tissues are only localized in the caudal segment of the mesencephalic nucleus [22]. Some anatomical textbooks state that the mesencephalic nucleus also contains signaling from oculomotor muscles and receptors from TMJ [18]; however, this theory has been repeatedly falsified as these pseudounipolar neurons are demonstrably located in the trigeminal ganglion, not in the mesencephalic nucleus [33,34,35]. The axons of these cells end mainly in the brainstem reticular formation (parvocellular part), in the trigeminal motor nucleus (glutamatergic signals), in the supratrigeminal nucleus and orexinergic hypothalamic nuclei [30,36]. Noteworthy is its projection to the trigeminocervical nucleus and from there, to motor centers of cervical muscles, beside other functions, important for mastication, typically for activation of suprahyoid and infrahyoid muscles during caudal movement (depression) of the mandible [36]. Lateral to the mesencephalic nucleus, are afferent and efferent fibers from the mesencephalic trigeminal tract (*tractus mesencephalicus nervi trigemini*). The mesencephalic nucleus is important for jaw-jerk (masseteric) reflex, as shown by experimental [37] or human studies [38]. However, the role of this nucleus in mastication is not completely elucidated; most likely, it provides the feedback for the masticatory apparatus (stomatognathic system), based on information from proprioceptors of masticatory muscles and mechanoreceptors from the periodontium. If the mesencephalic nucleus is lesioned unilaterally, thus removing all feedback from ipsilateral muscle spindles and important parts of the periodontal mechanoreceptors, experimental animals tend to chew on the contralateral side (they “feel” the food on this side); if the lesion is bilateral, they chew normally on both sides [37]. It remains an interesting result, which reveals that chewing is technically possible without proprioceptive signaling from masticatory muscles [37,39]. In this context, the finding that proprioceptive input does not play a major role in the differentiation of bite force is also important [40]. It was confirmed with findings in patients with lost afferents to mesencephalic nucleus [38]. It shows that proprioceptive signaling is important mainly for teaching of masticatory cycle; moreover, once established, it temporarily becomes less important—until the moment, when a new situation appears and establishment of a new masticatory pattern is necessary, such as after the increase in the vertical dimension of occlusion or loss of the so-called “supporting zones of Eichner” [39,41]. Conversely, experimental work showed that for the regulation of jaw elevation force, the mechanoreceptive signaling from periodontal tissues is necessary [40]. This mechanism protects the teeth against damage (infraction, fracture), as numerous clinical experiences with dental implants (having no periodontal mechanoreceptors) have repeatedly shown [42]. Moreover, the mesencephalic nucleus remains a crucial neural structure in parafunctional orofacial clinical entities such as bruxism (for review, see article by Giovanni and Giorgia [30]).

#### 4.2.4. Motor Trigeminal Nucleus

Motor trigeminal nucleus (*nucleus motorius nervi trigemini*) is a somatomotor nucleus located under the rostral third of the rhomboid fossa. Its fibers form the motor part of the trigeminal nerve, located in the mandibular nerve. It innervates all masticatory muscles (masseter, temporalis, medial and lateral pterygoid) as well as mylohyoid, anterior belly of digastric, tensor tympani, and tensor veli palatini. Cortical projections to the motor trigeminal nucleus are crossed as well as uncrossed; thus, unilateral lesion of this nucleus does not cause significant impairment of the masticatory cycle [18].

### 4.3. Temporomandibular Joint

TMJ is a compound, paired (bicondylar), and pivoting hinge joint developed between the mandibular (glenoid) fossa and the articular eminence (*tuberculum articulare*) of the temporal bone and mandibular condyle. Between the joint, the articulating disc (*discus articularis*) is inserted. This special and unique biconcave fibrous cartilage divides the joint cavity into the superior discotemporal and inferior discomandibular parts. The articular disc works as a shock absorber as well as a tensile force absorber, forming the basis of convex-concave contact of articular facets [16,26]. Its main parts in the ventrodorsal direction are: (1) anterior attachment of the superior head of the lateral pterygoid muscle, (2) anterior band (anterior rim, anterior dense portion of the disc), (3) biconcave intermediate zone, (4) posterior band (posterior rim, posterior dense portion of the disc), and (5) bilaminar zone, composed of the superior and inferior retrodiscal laminae, which enclose the Zenker’s retroarticular fat pad. The main axis of the load of the disc runs through the midpart of its biconcave intermediate zone, where the ventrocranial part of the condyle and dorsocaudal part of the articular eminence are in contact—both parts are convex; hence, the biconcave disk remains the crucial structure for the transfer of convex-convex surfaces to two convex-concave surfaces [16,26].

#### Temporomandibular Function

Movement of TMJ is limited by many ligaments. Intraarticular (intracapsular) ligaments, which develop in the joint cavity include the anterior, medial, and lateral discal ligaments together with discotemporal (“Tanaka’s”) and discomalleoar (“Pinto’s”) ligaments. Ipsiarticular (“collateral“) ligaments, formed in proximity to the articular capsule, include the temporomandibular (lateral) and medial ligaments. Extraarticular ligaments, formed at a distance from the articular capsule include sphenomandibular, stylomandibular, pterygomandibular ligaments, and *tractus angularis*. Of particular clinical interest are discomalleoar (Pinto’s) and malleomandibular ligaments (part of the sphenomandibular ligament), which connect the structures of the middle ear and the mandibular condyle or articulating disc, thus forming a morphological background for the development of tinnitus in patients with functional problems of TMJ, in particular those of the cervico-cranio-mandibular system [43,44].

Importantly, the position of the patient’s head is identical to that of the mandibular fossae of TMJ [45]. Thus, any change in alignment of the cervical vertebrae immediately affects the alignment of TMJ structures and their function. For example, if the C_2_ vertebra is rotated to the left, the head (together with the mandibular fossae) rotates to the right around the ventrodorsal axis passing through the *glabella* [46]. Accordingly, the bipupilar line (transverse line connecting pupillae of both eyes) is inclined to the right; thus, the right eye (and the right mandibular fossa) is positioned slightly downwards than the left eye (and the left mandibular fossa). A patient may not be aware of this change because the central visual cortex and visual connectomes “re-count” the information such as the head would be in a perfectly horizontal position (for review see Wei, 2018 [47]). This vertebro-cranio-mandibular relation has been repeatedly verified both in experimental animals [48,49] and human patients [50,51,52,53,54]. Elegant experimental study has been provided by D’Attilio et al. [48], who investigated the impact of occlusal interference on the spine of rats creating composite interferences on the right molar of each rat in the experimental group, observing that all such rats developed scoliotic curvature. After creating a composite interference on the opposite side as well (rebalancing of the occlusion), 83% of the rats in the experimental group had their vertebral curvature restored [48]. Moreover, in experimental animals with unilateral occlusal interference, condylar bone resorption of the contralateral side has been observed 1 week later [49]. This finding has been directly and indirectly verified in human patients [50,51,55,56,57,58,59,60] and the amount of scientific evidence of this phenomenon is rapidly increasing. Of note, when such conditions occur, a change in the occlusal (axilla-mandibular) relationship often leads to misalignment of teeth (malocclusion). The more slowly the cranio-cervical relationship change develops, the greater is the natural tendency to align the teeth (dentoalveolar compensatory mechanism), provided by efficient remodeling capacity of complex periodontal structures. This mechanism is significantly more efficient in growing children than in adults [61]. If this mechanism, and increased attrition/abrasion of the teeth is insufficient, particular teeth (or dental work such as crowns or bridges) are overloaded (traumatic articulation/occlusal trauma) and can suffer from periodontal breakdown and fractures. Moreover, if this malocclusion is fixed, such as in a case of functional unilateral posterior crossbite, it increases the discrepancy of maximum intercuspation and temporomandibular centric relation to pathologic level, which, if not treated properly, can affect further growth and development of the jaws and occlusion, leading to significant skeletal asymmetry, which may require surgical correction [50,51,62]. Moreover, concomitant abnormal mandibular movements may lead to adverse effects both on TMJ and other structures of the stomatognathic system, causing pain and various symptoms [50,51,63,64].

## 5. Integrative Function of the Cervico-Cranial Complex

The basic control of jaw-opening and jaw-closing is provided by a set of jaw reflexes (trigemino-trigeminal reflexes). The jaw-closers (masseter, temporalis, and pterygoid muscles) serve to close the jaw (mandibular elevation) under normal circumstances and to open it (mandibular depression) when they undergo inhibition. The jaw-closers are excited by A-alpha muscle spindle input and strongly inhibited by A-beta encapsulated mechanoreceptors and possibly A-delta free nerve endings [65]. Uniquely, among the primary sensory neurons, these afferents have their cell bodies in the CNS, in the mesencephalic trigeminal nucleus (see above), rather than in the ganglion, as is typical for other afferents. Moreover, short collaterals connect monosynaptically with synergistic jaw-closing motoneurons in the pontine trigeminal motor nucleus; however, no collaterals cross the midline [65]. Importantly, the excitability of jaw reflex interneurons and primary afferent terminals is controlled during mastication in a way that the sensory detection threshold rises during movement, with a significant change in the perception of oral stimuli during mastication [32].

The rhythmic orofacial movements produced during mastication require the coordination of several jaw, facial, hyoid, and tongue muscles. The basic pattern of rhythmic jaw movements produced during mastication is generated by a neuronal network located in the brainstem and referred to as the masticatory **central pattern generator**. This network, which is composed of neurons mostly associated to the trigeminal system, is found between the rostral borders of the trigeminal motor and facial nuclei [66]. It is capable of the production of rhythmic network activity both and/or without rhythmic inputs from descending or sensory afferents. However, sensory feedback, particularly that from intraoral mechanoreceptors, modifies the basic pattern and is particularly important for the proper coordination of the tongue, lips, and jaws [32]. The central pattern of mastication is generated in two stages: the rhythm, provided by neurons in the midline reticular formation, and the bursts, provided by premotor neurons near the brainstem motor nuclei. The burst generators excite the mandibular opener alpha-motoneurons and inhibit the mandibular closers during the opening phase; however, during closing, the opener motoneurons are not inhibited. Two types of gamma-motoneurons are also involved: dynamic gamma-motoneurons are tonically active during mastication, while static gamma-motoneurons are excited during mandibular closure [32]. The adult masticatory pattern is established at different ages; it varies considerably among individuals [66]. Although mastication is often considered to be stereotyped, there is much variability from cycle to cycle. An older, although important, study of Dellow and Lund showed that the basic pattern could be generated by the brainstem alone in a decerebrated, paralyzed animal [67]. However, the central pattern generator is affected (feedbacked) by many other centers, such as the cortex (including kinesiotopic representation in cortical masticatory area in the most inferior part of the precentral gyrus; via ipsilateral and mainly contralateral corticobulbar tracts), amygdala, hypothalamus, anterior pretectal nucleus, red nucleus, periaqueductal gray, brainstem reticular formation (lesioning of the medial reticular nuclei abolishes mastication), cerebellum or various parts of basal nuclei (for review see Morquette et al., 2012; Lund, 1991 [32,66]). Moreover, inputs from these areas have primarily modulatory functions and are not required to generate the basic masticatory movements that are produced by activation of the masticatory central pattern generator, as shown on decerebrate animals and other models [32,66,68]. However, they are essential for the adaptation of the mandibular movement to the hardness of the food, to compensate for unexpected perturbations and many other delicate masticatory and mastication-related functions [32,66,69]. The central pattern generator also modulates primary afferents and interneurons to suppress unwanted reflexes and favor those that enhance motor performance [32]. More detailed information on the central pattern generator function is available in the literature [32,65,68], but it extends beyond the focus of this review.

The masseter, temporalis, and both medial and lateral pterygoid muscles are often termed the masticatory muscles (muscles of mastication). However, other muscles are also involved in mastication, and are not less important. Due to their position, i.e., attachment from the inferior of the mandible, these muscles can be called **inframandibular masticatory muscles**. Muscles directly attached to the mandible include the anterior belly of the digastric and the mylohyoid. Indirectly attached muscles include platysma, all infrahyoid muscles (sternothyroid, sternohyoid, thyrohyoid, omohyoid), stylohyoid, posterior belly of digastric, pharyngeal constrictors, buccinator, and orbicularis oris. From these, the infrahyoid muscles, innervated by the deep cervical ansa (cervical spinal nerves C_1_–C_3_), are of particular importance for COP, due to their activation caused by C_1_–C_3_ spinal nerve irritation. The temporomandibular symptoms including OP appear because inframandibular muscles hold the position of the mandible in perfect midline, but the fossae are positioned incorrectly; thus, the alignment of particular structures of TMJ is incorrect [45,70,71]. Importantly, a significant number of muscle spindles have been found in mandibular elevators, but not in mandibular depressors [32,66]. Thus, the tendency to correct the mandibular position comes mainly from mandibular elevators, thus increasing the possibility of tooth gnashing, grinding, clenching, bracing, thrusting, tapping, and bruxing, together forming a wide range of symptoms of bruxism (for review, see article by Giovanni and Giorgia [30]). Moreover, muscle spindle afferents show various patterns of activity during mastication, ranging from excitation only during mandibular opening to their strong fire during slow closure, thus responding to fusimotor drive [32,37]. Increasing number of data show that the CNS receives several types of feedback signals enabling it to control muscles of mastication in particular movements, both gross and very fine [32,66]. During mastication, trigeminal ganglion neurons provide positive feedback to jaw-closing motoneurons, because heavy pressures generated during the jaw-closing phase of mastication cause the jaw-closing phase to lengthen and jaw-closing motoneurons to fire at higher frequencies [66], leading to a vicious cycle.

Importantly, muscle chains are developed among the particular bony structures. For example, the cervical spine (and occipital bone via pharyngeal raphe attachment) is connected with the anterior mandible (chin) via a muscle chain composed of the superior pharyngeal constrictor muscle, pterygomandibular raphe, buccinator muscle, and inferior part of the orbicularis oris muscle. Dorsally, this muscle chain is connected with the anterior periosteum of the cervical vertebrae via small, but firm pharyngovertebral ligaments. Anteriorly, the orbicularis oris is attached to the periosteum of the anterior mandible. Spasm or any other functional impairment in this muscle chain involves the growth (in children) and movements of the mandible (in all patients), and mandibular protrusion in particular. The involvement of these muscle chains must be considered in differential diagnosis as well as during treatment.

Neurologically, the impairment/loss of occlusal harmony and the development of occlusal interferences significantly impair the execution of jaw reflexes, as well as the function of masticatory central pattern generator in the brain, thus impairing mastication, vital for the preparation of food for digestion by breaking it down into pieces that can be swallowed [66].

## 6. Novel Concepts of Functional Synthesis of Cervico-Cranial Interactions

Based on scientific evidence and our clinical experience, we recently developed two concepts of OP etiopathogenesis and management, which we present for the first time in this review.

### 6.1. Rocabado Tricentric Concept of Mouth Sensorimotor Control

**Figure 2:**
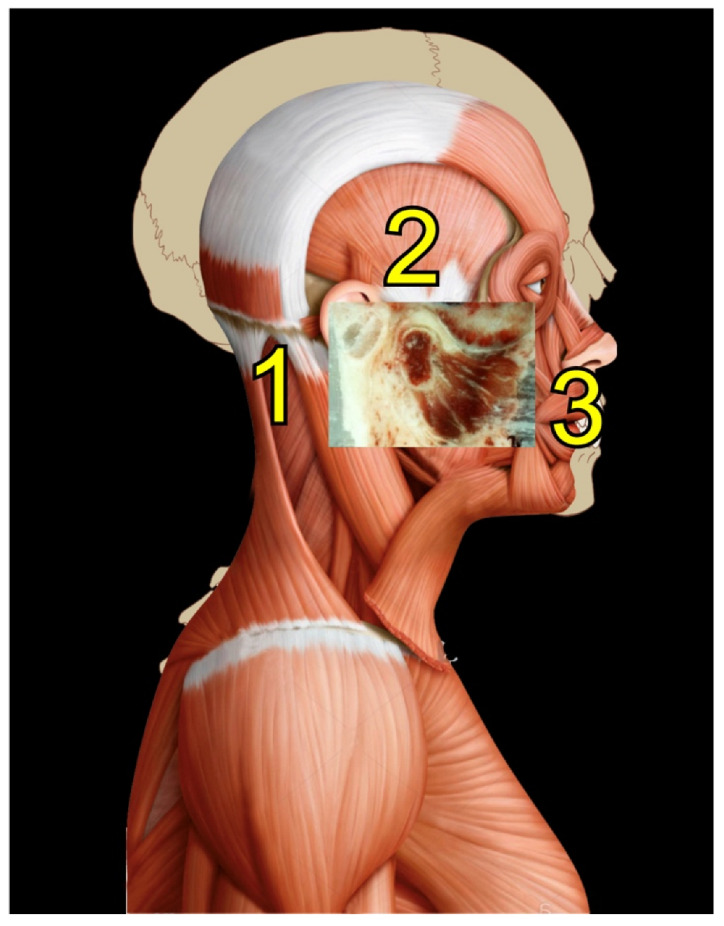
Rocabado tricentric concept. Source: Rocabado Institute, Chile. Craniovertebral, cranio-mandibular, and centric occlusion at rest are all coordinated to allow functional masticatory system and physiology of the mouth motor and sensory control. This automatic, dynamic mechanism will facilitate a stable fossa-condyle long-lasting congruent joint surface relation.

**Figure 3:**
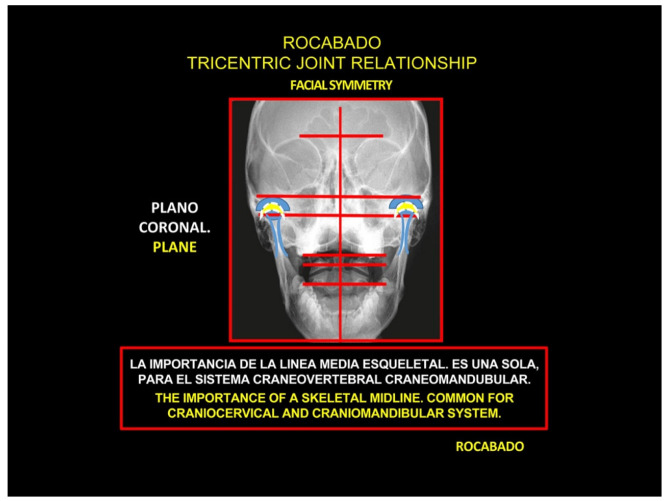
Rocabado tricentric concept.

Rocabado tricentric concept incorporates (1) craniovertebral, (2) cranio-mandibular, and (3) centric occlusion at rest, all mutually coordinated to allow proper masticatory function and mouth sensory and motor control (Figure 2 and Figure 3). The automatic and dynamic interactions of the three interconnected systems allow for a stable and lasting relation of the mandibular condyle joint facets, functional masticatory system, and physiology of the mouth motor and sensory control. This automatic dynamic mechanism allows for a stable, fossa-condyle, long-lasting, congruent joint-surface relation.

#### The Importance of the Skeletal Midline

Physiologically, the mechanism of COP is analogous to the pain felt in the shoulders, chest wall, buttocks, or lower limbs, which is referred from proximal spinal sources; hence its familiarity with pain specialists [5]. The trigeminocervical convergence allows for pain arising from the upper cervical nerves to be referred to regions of the head innervated by trigeminal afferents, such as the orbital, frontal, and parietal regions [4,5,23,72]. Such referral patterns have been elicited in healthy volunteers by experimental noxious stimulation of cervical structures targeting suboccipital and posterior cervical muscles [73,74]. Noxious stimulation of more rostral structures in the cervical spine elicited referred pain in the occipital region, as well as more distant frontal and orbital regions. Conversely, the stimulation of more caudal spine structures elicited pain in the neck radiating to the occipital regions, although not to the anterior regions of the head. It was subsequently shown that noxious stimulation of the atlanto-occipital and lateral atlanto-axial joints, C_2_–C_3_ zygapophysial joint, and C_2_–C_3_ intervertebral disc can produce pain in the occipital region [5,75,76,77,78].

COP must demonstrate a temporal relationship with the cervical disorder to support causality [10]. Cervical range of motion may be reduced, and headache can worsen with particular movements and/or provocation maneuvers of the neck. The demonstration of a cervical disorder on imaging may be supportive of a diagnosis of cervicogenic pain but does not establish firm evidence of causation. Thus, causation should be considered in the context of the clinical presentation and suspected underlying disorder. Tumors, fractures, infections, cervical spondylosis, osteochondritis, and rheumatoid arthritis of the spine have not been formally validated as causes for OP, but may support causation for COP in certain individual cases. Due to the convergence of cervical and trigeminal nociception, upper cervical myelopathy (C_1_, C_2_, C_3_) may be causal for OP [10,12,79].

**Figure 4:**
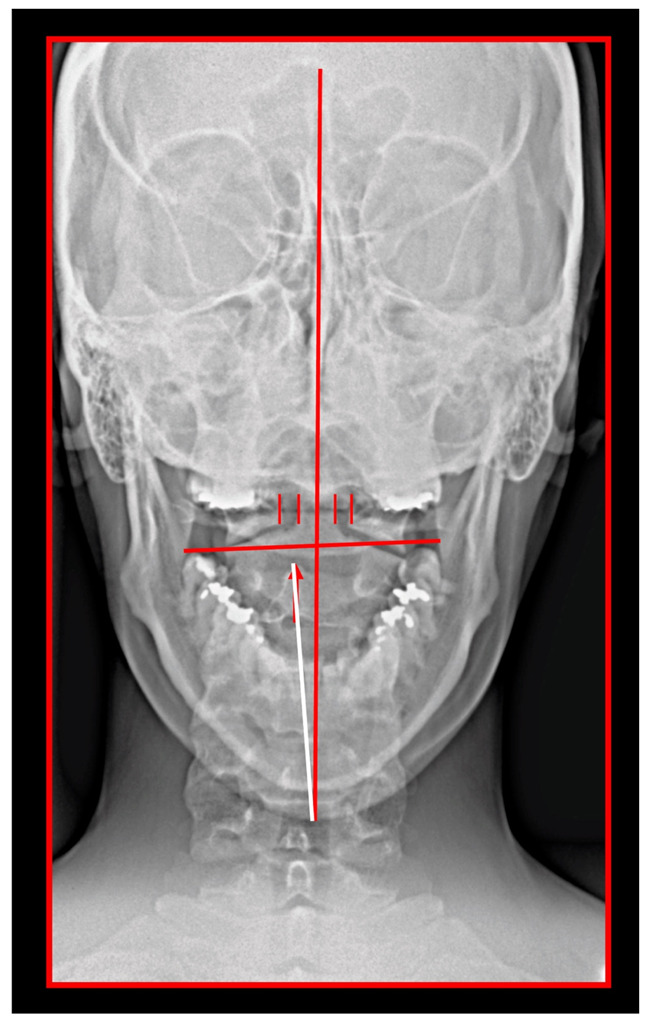
Transoral *atlas*-*axis* X-ray. Skeletal midline—*dens axis*—spinous process of C_2_ should be in one single line. In this patient, spinous process of *axis* is deviated to the right; hence, the *axis* is rotated to the left. The position of the *atlas* is measured from the lateral mass of the *atlas* to the *dens axis*. The space is increased on the right; hence, the *atlas* is rotated to the right. The mandibular occlusal plane is inclined. The cranium is rotated to the left; the left eye pupil is lower than the right.

Misalignment at the cranio-cervical junction may also play a pathogenic role (Figure 4). Complementary studies have mapped the distribution of referred pain related to the atlanto-axial and zygapophysial (facet) joints of the upper cervical spine [10]. Patients with pain from a particular joint do not experience exactly the same distribution of pain, but there are similarities in the distribution [5]. Misalignment of the first cervical segment can cause impingement of the first two cervical nerve roots (C_1_, C_2_) as they exit the spine. Stimulation at the C_1_ level experimentally evoked occipital or cervical pain in those without migraine, although it was more likely to evoke periorbital and frontal pain in patients with a history of migraine [80]. Moreover, C_2_ and C_3_ stimulation refers pain to the occipital or cervical region [81]. Although studies have not supported middle- or lower-cervical lesions (below C_4_) as being contributory to OP, anastomosis between the spino-cervico-thalamic tract and trigemini-cervical complex may support this possibility [81]. These data show that the structures capable of producing referred pain to the head are those innervated by the C_1_, C_2_, and C_3_ spinal nerves. No experimental studies have shown that structures innervated by lower cervical nerves are capable of directly causing headaches [5]. However, intermediate mechanisms, such as muscle tension and secondary kinematic abnormalities that affect the upper cervical joints may be involved [5,82].

The relationship to body posture is critical. Many studies have provided evidence of a direct connection between body posture and vertebro-cranio-mandibular system [50,51,55,56,57,58,59,60]. For example, in children with unilateral crossbite, increased occurrence of postural problems such as oblique shoulder, scoliosis, oblique pelvis, and functional leg length differences have been observed [55]. 

Faulty body posture and head positioning may result in structural muscle-tissue changes. The deep cervical flexors may undergo a reduction in the number of type I fibers due to muscle inhibition from chronic upper cervical posterior rotation. This, in turn, may help explain the deficits in endurance of the deep cervical flexors observed in patients with COP. According to Watson and Trott, the upper cervical flexors provide a “holding mechanism” for balance and stability of the head [83]. Consequently, the upper or deep cervical flexors have a higher proportion of type I (slow-twitch) versus type II (fast-twitch) fibers, which renders these muscles more resistant to fatigue under normal circumstances. Thus, the importance of posture will be stressed in the following text, as well.

### 6.2. Concept of Cervical Origin of Bruxism

Bruxism represents a complex of severe clinical symptoms, defined by the American Academy of Orofacial pain as “total parafunctional daily or nightly activity that includes grinding, gnashing, or clenching of the teeth”. It occurs in the absence of subjective consciousness, and it can be diagnosed by the presence of tooth-wear facets that have not resulted from the chewing function [84]. There are numerous articles and even comprehensive books focused on bruxism (e.g., Paesani, 2010 [85]). Interestingly, only a few studies and reviews focus on muscles, although they undoubtedly represent a very important factor in the etiopathogenesis of bruxism—simply and concisely, “without muscles, there is no bruxism”. Conversely, majority of studies focus on the teeth, where the consequences (but not the etiopathogenesis) are identifiable. Moreover, in the picture of the above mentioned cranio-orofacial, neuro-orthopedic connections, nervous system involvement should be suspected in aspects other than only psychologico-psychiatric facet, as often made by experimenters and clinicians. Furthermore, studies reveal not causation, but a correlation of psychologico-psychiatric aspects and bruxism, indicating they represents mainly secondary, not primary etiological factor in bruxism [86,87,88]. From our clinical experience, a patient with OP suffering from a primary psychiatric disorder is extremely rare; however, most of these patients have secondary psychological impairments (Šedý, Rocabado, unpublished observation). The crucial factor is time—the proper diagnosis of such patients is often difficult, demanding and time-consuming; contrarily—immediate referring such patients for psychological/psychiatric examination is the opposite. Psychological and psychiatric problems, among the most important seem to be anxiety and depression, thus rather reveal and/or further increase the severity of present bruxism, caused primarily by other (primary) etiopathogenic factors [86,87,88,89].

Our concept proposes a chronic irritation of the C_1_–C_3_ spinal nerves by the improper alignment of the cervical vertebrae and/or degenerative changes in particular vertebrae in close relation to the intervertebral foramina, causing repeated activation of the infrahyoid muscles via branches of the deep cervical ansa (*ansa cervicalis profunda*). These repeated contractions of infrahyoid muscles cause the repetitive minute depression of the hyoid bone as well as the mandible (via the suprahyoid muscles). In the opposite direction, the supramandibular chewing muscles, mainly the mandibular elevators (masseter, temporalis, medial pterygoid) function to reestablish the so-called rest position of the mandible (resting position of the mandible, clinical rest position, rest vertical dimension, mandibular postural position, physiologic rest position, mandibular rest position, vertical dimension of rest) via central pattern generator in the CNS (see above), thus causing sustained muscle contraction leading to bruxism symptomatology, which becomes progressively severe with time.

## 7. Clinical Syndromes in Cervicogenic Orofacial Pain

Many clinical syndromes have been described in COP. In this review, we describe the ones most important for clinical use in daily practice.

### 7.1. Cervical Spondylosis

Cervical spondylosis as a primary source of referred OP has been considered important and clinically relevant for decades. Franks in 1968 analyzed 951 patients referred to TMJ specialists during a 5-year period, finding that the problems in 23 were primarily caused by cervical spondylosis [90]. Since then, many studies have identified a cervical origin of orofacial/temporomandibular pain and other symptoms, as detailed in other parts of this review.

### 7.2. Occipital Neuralgia

The incidence of occipital neuralgia was reported to be 1.8% of headaches [91]. It is caused by irritation of the greater or lesser occipital nerve, characterized by paroxysmal shooting or stabbing pain over the posterior scalp, in the distribution of the occipital nerve. Due to interneural connections in the trigeminal spinal nuclei through the trigeminocervical complex, pain from occipital neuralgia may be referred to the ipsilateral temporal, frontal, or orbital areas [10]. 

Pain from occipital neuralgia typically irradiates from the suboccipital region towards the vertex and is unilateral in 85% of patients [10]. The pain may be severe, and paroxysmal attacks may last from seconds to minutes. Between paroxysms, there may be a persistent dull ache over the occipital nerve territory, as well as corresponding dysesthesia or allodynia. Continuous occipital pain in the absence of any associated dysesthesia or allodynia should raise suspicion for possible referral of pain from the cervical structures. There is typically tenderness over the affected nerve branches and there may be trigger-point tenderness at the emergence of the greater occipital nerve or in the C_2_ distribution. Tingling may also be evoked by light pressure or percussion over the nerve, known as Tinel’s sign. Pain with hyperextension or rotation of the neck when patients are in bed may also be a feature, known as the pillow sign [10,92]. Occipital neuralgia must be carefully differentiated from pain in the occiput referred from the atlanto-axial or upper zygapophyseal joints, which should be more appropriately diagnosed as cervicogenic pain (for review, see Barmherzig and Kingston, 2019 [10]).

Importantly, Bogduk and Govind recognized occipital neuralgia as an outdated diagnosis, used before the concept of somatic referred pain was widely understood, when physicians believed that any pain in a particular region was due to some affliction of the nerve that ran through that region. They argued that the proposition that the greater occipital nerve could be compressed between the posterior arch of the *atlas* and the lamina of the *axis* was incompatible with the anatomy and biomechanics of those vertebrae, and that deep aching occipital pain was more likely to be somatic referred pain from an upper cervical joint [5].

### 7.3. Ponticulus Posticus Syndrome

Any shift/misalignment of the *atlas* can cause a direct impingement of the vertebral artery as it passes through the transverse sulcus or foramen, thus plausibly compromising blood flow to the vertebrobasilar system. One of the most common sources of vertebrobasilar insufficiency, which can also impair the first spinal nerve, is *ponticulus posticus* (Kimmerle anomaly), an artificial bony emergence, crossing the vertebral sulcus of the *atlas*. Through this region, the V_3_ part of the vertebral artery is running, together with the abovementioned first cervical nerve and sympathetic as well as venous vertebral plexus [93]. After the calcification of the ligament crossing the vertebral sulcus, an artificial arcuate foramen is formed. It is not a novel finding; although it was first described by W. Allen in 1879 [94], it has not been recognized as a source of COP and/or vertebrobasilar insufficiency until recently. If symptomatic, it represents a morphological basis of tunnel syndrome. A meta-analytic study of 55,985 cases published between 1885–2015 found incomplete *ponticulus posticus* in 13.6% of the population and complete *ponticulus posticus* in 9.1% [95]. In 53.1% of cases, it was bilateral, complete in 59%, and incomplete in the remaining 41% [95]. It occurred more often in North Americans (11.3%) and Europeans (11.2%), and less often in Chinese (4.4%) [95]. Complete *ponticulus posticus* was more frequent in men (10.4%), whereas the incomplete occurred more frequently in women (18.5%) [95]. It more frequently occurs in patients with Gorlin-Gotz syndrome [96]. Morphometric analysis reveals that its mean horizontal diameter (width) is 5.65 mm (range 5.29–5.83 mm) and its mean vertical diameter (height) is 5.16 mm (range 4.86–5.46 mm) [95]. The manifestations of this tunnel syndrome include vertigo, migraine, and Barré-Lieou syndrome, among others. Vertigo is caused by compression of the vertebral artery during head movements, i.e., the movements of the atlanto-occipital joint [95,97,98]. Migraines correspond to COP caused by trigeminocervical convergence, due to irritation of the first cervical spinal nerve [95]. However, the compression of the vertebral artery may also be etiopatogenically involved [98]. Moreover, we must consider that spinal nerves are supplied by branches of the vertebral artery [18]. The Barré-Lieou syndrome represents a combination of headache, nausea, retro-orbital pain, problems with phonation, and visual problems and is clearly associated with *ponticulus posticus* [95,97]. Complete *ponticulus posticus* is 5–11 times more likely to compress the vertebral artery than incomplete *ponticulus posticus* [95]. Regarding diagnosis, computed tomography (CT) has a 20% higher success in uncovering its presence than lateral radiography of the cervical spine [95]. Importantly, the presence of *ponticulus posticus* remains the morphological limitation of conservative treatment; in case of conservative treatment failure, it may be necessary to indicate its surgical removal [95,97,98].

### 7.4. Triggered Pain

Chronic or persistent pain conditions represent one of the most common causes of disability worldwide. Trigger points are specific sites in muscles, located within taut bands, which are discrete bands of contracture muscle fibers that can be palpated and visualized by particular imaging methods (for review, see Travell and Simons, 2013 [99]. They have greater than normal degree of stiffness than that of normal muscle tissue [100]. They develop most often due to muscle overload, i.e., when an applied load exceeds the capability of the muscle to respond adequately, particularly following unusual or excessive eccentric or concentric loading [101]. Myofascial trigger points in the muscles of the neck can refer pain to the face and head, and vice versa, although less often. Travell and Simons have stated that myofascial trigger points may develop from structural inadequacies, postural stress, and constriction of muscles. At the cranio-cervical junction, the structural inadequacy may be represented by a chronic *atlas* (C_1_ vertebra) misalignment that generates mechanical stress, which then leads to the formation of trigger points [99].

### 7.5. Atypical Facial (Oro-Facial) Pain

Atypical facial pain (AFP), or persistent idiopathic facial pain is a well-recognized syndrome, where the depression or psychosomatic causes are mostly suspected as primary etiological factors. It is a chronic and diffuse distribution of facial pain along the distribution of the trigeminal nerve. This condition occurs in the absence of any neurologic deficit or known etiology. It presents mostly with neck-muscle tension and OP. Despite the limitations of evidence-based literature, tricyclic antidepressants such as amitriptyline or nortriptyline have proven effective and are considered the treatment of choice for AFP [102,103]. However, AFP is one of the most challenging conditions to diagnose due to the lack of clear diagnostic criteria, as well as specific, evidence-based guidelines for management. This condition is diagnosed mostly by the exclusion of other known etiologies. Specific disease modalities cannot be targeted, resulting in a deficiency of a clear treatment protocol. Thus, this diagnostic category is often questioned [103,104]. Based on our empirical clinical experience, we strongly suspect that the diagnosis of AFP is often, but not always, used for patients with unrecognized COP (Šedý, Rocabado, unpublished data).

### 7.6. Pain Due to Iatrogenic Causes

COP can be caused by iatrogenic factors. Typically, it occurs when the vertical dimension of occlusion drastically increases, typically due to a massive occlusal splint. Such splints are sometimes fabricated for conservative treatment of obstructive sleep apnea, to open upper airways during sleep (for review see Marklund et al., 2019 [105]). However, this extreme posteriorotation of the mandible leads to compensatory posteriorotation of the head, leading to enhancement of cervical lordosis (“hyperlordosis”) causing the compression of C_1_–C_3_ spinal nerves, leading to COP.

## 8. Diagnosis of Cervicogenic Orofacial Pain

Proper diagnosis of COP remains a crucial part of clinical protocol. Although it can be time-consuming, demanding, and complex, it presents a fundamental take-off step for successful curative treatment of COP.

### 8.1. Diagnostic Criteria

Antonaci et al. proposed seven criteria for cervicogenic headache: (1) Unilateral headache without side-shift, (2) symptoms and signs of neck involvement: pain triggered by neck movement or sustained awkward posture and/or external pressure of the posterior neck or occipital region; ipsilateral neck, shoulder, and arm pain; reduced range of motion, (3) pain episodes of varying duration or fluctuating continuous pain, (4) Moderate, non-excruciating pain, usually of a non-throbbing nature, (5) pain starting in the neck, spreading to oculo-fronto-temporal areas, (6) anesthetic blockades abolish the pain transiently provided complete anesthesia is obtained, or occurrence of sustained neck trauma shortly before onset [106]. Satisfying criteria 1 and 5 qualify for a diagnosis of possible cervicogenic headache. Satisfying any additional three criteria advances the diagnosis to a probable cervicogenic headache [106].

### 8.2. Importance of Prompt and Accurate Diagosis

Undiagnosed and unaddressed pain can be refractory, leading to disability, and reduction of quality of life [10]. When deep knowledge and clinical experience of the medical specialist is set aside, the most important factor for a proper diagnosis is the time spent with the patient. Patience is important for obtaining a detailed and valid and reliable medical history of the patient (anamnesis). For COP, the most reliable features are previous neck trauma, pain that starts in the neck and radiates to the fronto-temporal region, pain that radiates to the ipsilateral shoulder and arm, and provocation of pain by neck movement [106,107,108]. 

**Figure 5:**
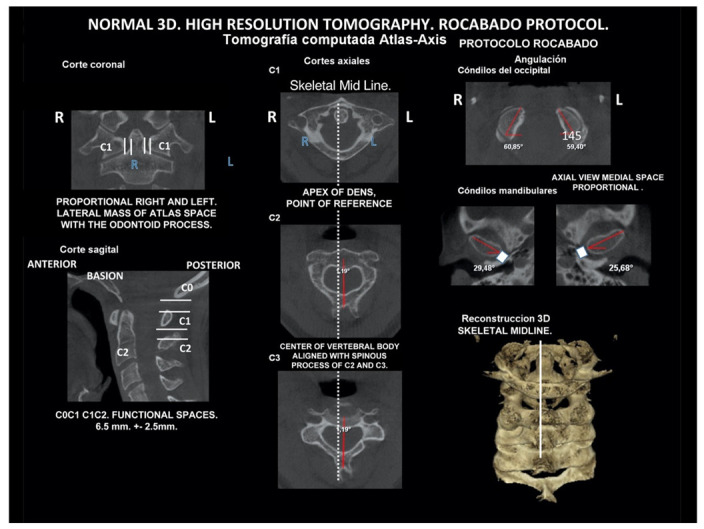
Rocabado diagnostic protocol for cervical spine.

These patients are often frustrated from previous “improper examination/treatment failure” vicious cycles. Although they are highly motivated for treatment, their confidence in medical authorities and proposed treatment methods may be low. In addition, most psychological signs are not primary (i.e., primary psychiatric diseases such as schizophrenia or endogenic depression) but secondary, caused by severe impairment of their psychosocial aspects of life caused by long-term debilitating pain and other orofacial disabilities [109,110]. The following detailed clinical examination is also necessary. Additional maneuvers on physical examination should include movement tests of the cervical spine, such as passive flexion, extension, and rotation; segmental palpation of the cervical facet joints, and; it should also include the assessment for palpation tenderness over the greater-occipital nerve, lesser-occipital nerve and upper-cervical muscle groups [10,16,111] (Figure 5).

### 8.3. Local Anesthetic Blockade as a Diagnostic Tool

COP may respond to local anesthetic blockade of the occipital nerves (greater, lesser, or both). While this intervention is sensitive, it is not specific. Many primary headache disorders including migraine, tension-type headache, and cluster headache may also demonstrate a similar response. Clinically, there may be a significant overlap in the clinical appearance between COP, occipital neuralgia, migraine, and tension-type headache with pericranial tenderness [10,79]. 

Neck pain can be a predominant feature in up to 68% of patients with migraine and tension-type headaches [79]. In the case of migraine, neck pain may occur as a prodromal symptom, intra-attack, or as a postdrome symptom [112]. Due to connections with the vestibulocochlear, glossopharyngeal, and vagus nerves, symptoms such as tinnitus, dizziness, and nausea may be features of both occipital neuralgia and COP [113]. However, these features are less prominent in COP as well as in occipital neuralgia than in migraine.

## 9. Differential Diagnosis

COP can mimic several diseases, the most clinically important of which are presented here.

### 9.1. Dissecting Aneurysms

Dissecting aneurysms of the vertebral or internal carotid arteries can present with neck pain and headache. They are indicated by the onset of cerebrovascular features, which typically emerge within 1–3 weeks. If this differential diagnosis is not considered, there is a risk of patients being treated with cervical manipulation, with fatal consequences due to aggravation of the aneurysm [5,114,115,116].

### 9.2. Lesions of the Posterior Cranial Fossa

They are also important, as the dura mater and vessels of the posterior fossa are innervated by the upper cervical nerves. These lesions are distinguished by the onset of neurological features or systemic illness, such as meningitis of the upper cervical spine or herpes zoster. Moreover, the tiny, but clinically, extremely important muscles of the suboccipital triangle have an anatomically defined connection of their epimysia with cervical- and lower-cranial dura mater [117]. Importantly, the activity of these muscles is directly connected with the change in the head position [118,119].

### 9.3. Migraine

**Figure 6:**
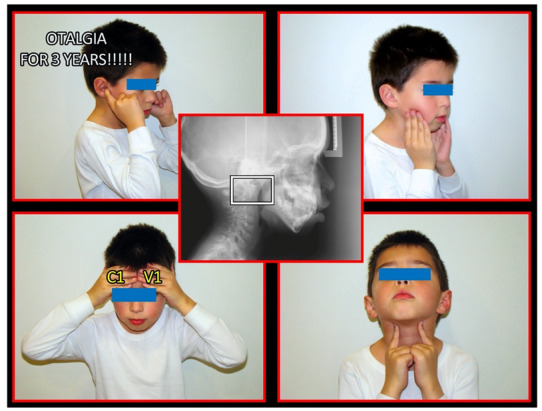
Pediatric patient with otalgia, headache, and pain of the occiput. Source: Rocabado Institute, Chile. *Atlas*-*axis* syndrome previously not diagnosed. The importance of the neurological connection between the cervical spine and the craniofacial and craniomandibular system. Patient showing sites of pain.

While migraine tends to have unilateral shifting, both COP and occipital neuralgia tend to be side-locked, and COP is often triggered by head movement (irritation of C_1_–C_3_ spinal nerves) (Figure 6). Pain radiation in COP tends to have a postero-anterior direction. However, in migraine, the pain tends to be more anterior, and radiation tends to be anterior-posterior. COP should also be differentiated from other secondary disorders such as headache attributed to cervical dystonia, Chiari malformation, cervical or vertebral artery dissection, whiplash injury, congenital malformations, space-occupying or destructive lesions, or infection [10,79,112,113,120]. 

### 9.4. Neck-Tongue Syndrome

Bogduk and Govind stressed that neck–tongue syndrome and C_2_ neuralgia can be confused with COP, because the C_2_ spinal nerve runs behind the lateral atlanto-axial joint and is accompanied by its dural sleeve and a substantial plexus of veins. Neck–tongue syndrome occurs when rapid turning of the head subluxates the lateral atlanto-axial joint posteriorly. Tension in the joint capsule causes ipsilateral occipital pain, while compression of the C_2_ spinal nerve produces numbness of the tongue [5,121,122]. 

### 9.5. Miscellaneous

Moreover, C_2_ compression/neuralgia can be caused by various disorders. Inflammatory disorders or injuries of the lateral atlanto-axial joint can result in the adjacent nerve becoming incorporated into the fibrotic changes of chronic inflammation [123,124]. The C_2_ spinal nerve can be compromised by a meningioma, neurinoma, anomalous vertebral arteries, and several other vascular anomalies [123,124,125,126,127,128]. Nerves affected by vascular abnormalities have several features indicative of neuropathy, such as myelin breakdown, chronic hemorrhage, axon degeneration and regeneration, and increased endoneurial and pericapsular connective tissue [125]. Unlike the dull, aching pain of COP, the features of C_2_ neuralgia are intermittent, lancinating pain in the occipital region associated with lacrimation and ciliary injection [5,123,125,128].

## 10. Diagnostic Use of Imaging Techniques

**Figure 7:**
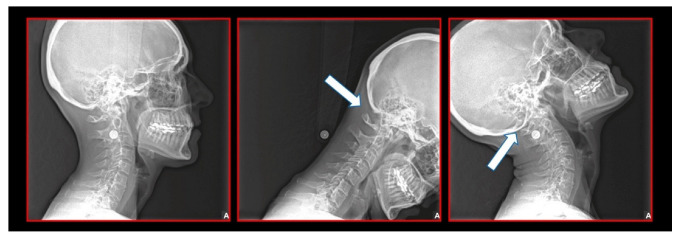
Normal lateral (sagittal) dynamic cephalometric analysis.

**Figure 8:**
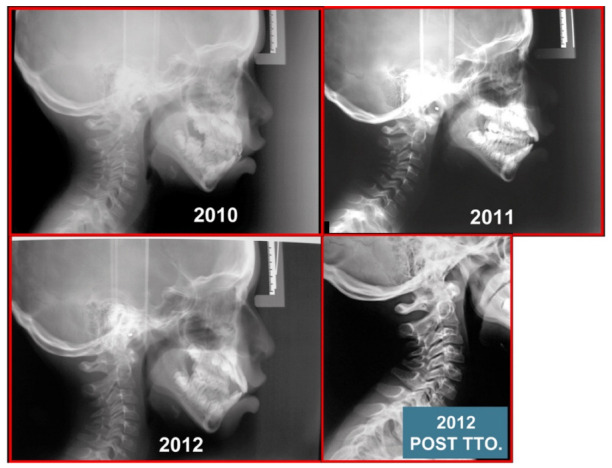
Pediatric patient with otalgia, headache, and facial pain of the occiput. *Atlas*-*axis* syndrome previously not diagnosed. A series of lateral cephalograms showing the treatment progress. There is the importance of the neurological connection between the cervical spine and craniofacial and craniomandibular systems.

**Figure 9:**
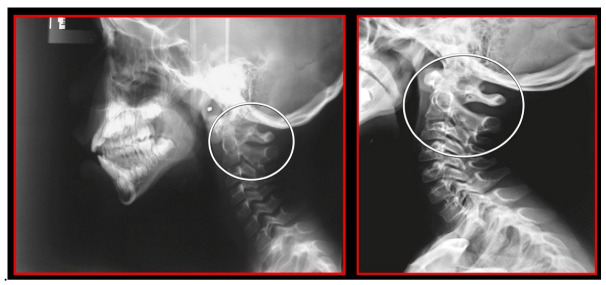
Rotation of the *atlas* on a lateral cephalometric radiograph. **Left:** Pre-treatment. Double ring of the *atlas* showing its rotation. Decreased functional spaces. **Right:** After orthopedic manual therapy. Only one posterior arch of the *atlas* showing the normalization of its rotation. Normalized functional spaces.

Several imaging methods have been proposed for the diagnosis of COP. However, in their indication, risk-benefit ratio needs to be evaluated in every case, to prevent their overindication (overtreatment). Moreover, analysis of previous medical specialist consultations (ear-nose-throat [ENT], neurological, dental, etc.) and results of previous examination methods (radiography, CT, magnetic resonance imaging [MRI], sonography, etc.) is also necessary. Imaging methods permit clinicians to objectively observe and confirm at least the seven most frequent positional faults among the occiput, *atlas*, *axis*, and C_3_ vertebra (Figure 7, Figure 8 and Figure 9). These positional faults induce craniovertebral intra-joint irritation, as well as capsular connective tissue, ligament soft tissue damage, tears, inflammation, bleeding, and muscle disorders such as shortening, tightness, spasm, adhesions with local or referred patterns of pain. These pathologies need to be addressed prior to reaching any definite therapeutical decision of occlusal contact changes during the process of oral rehabilitation. All dysfunctions are related to the trigeminal-cervical nucleus, affecting the occipito-atlanto-axial joints and synovial TMJ with a broad syndrome of head neck and facial pain with or without synovial TMJ disc pathology.

### 10.1. Neck X-ray

**Figure 10:**
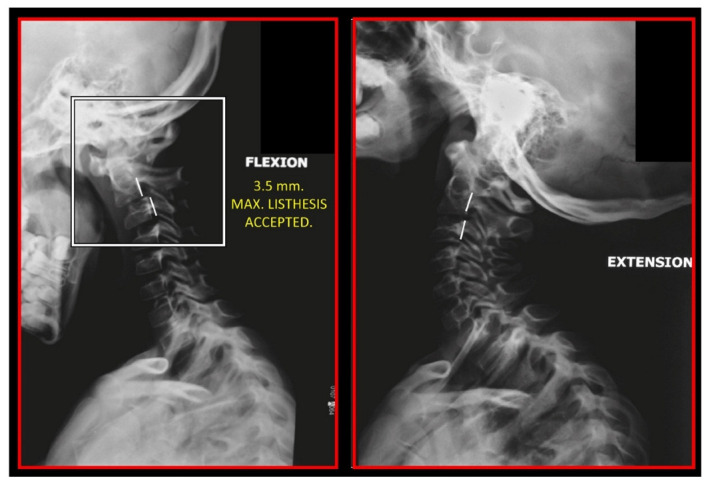
Static *atlas* on the occiput in flexion and extension. Additionally, notice the double ring of posterior arch of the *atlas* showing that it is also rotated and static. A 3.5-mm anterior listhesis of the *axis* on C3 during flexion. Lateral cephalometric study.

**Figure 11:**
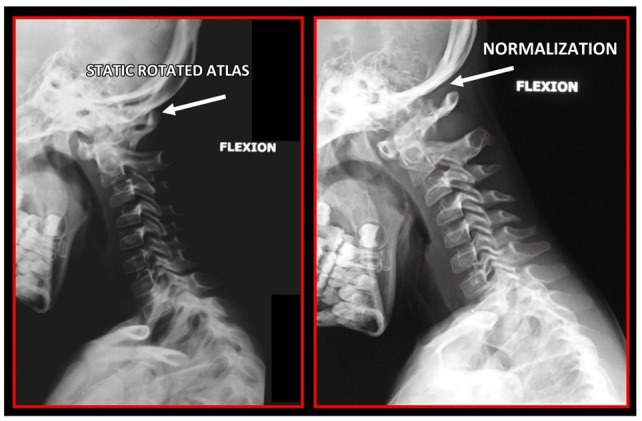
Rotation of the *atlas* on lateral cephalometric radiograph. **Left:** Pre-treatment. Double ring of the *atlas* showing its rotation. Decreased functional spaces. **Right:** After orthopedic manual therapy. Only one posterior arch of *atlas* showing normalization of *atlas* rotation. Normalized functional spaces.

**Figure 12:**
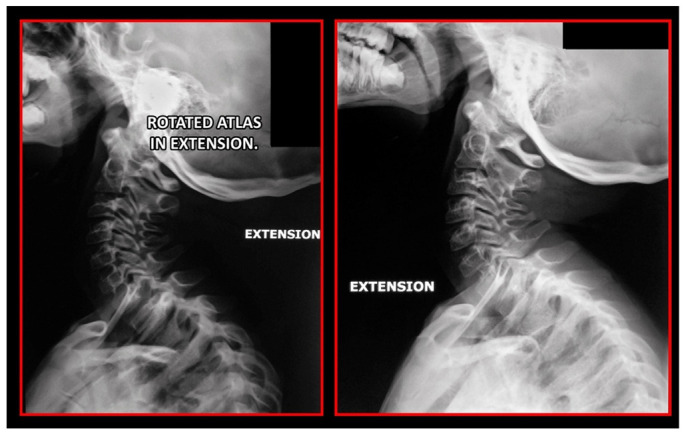
Rotation of the *atlas* on lateral cephalometric radiograph. **Left:** Pre-treatment. Double ring of the *atlas* showing its rotation. Decreased functional spaces. **Right:** After orthopedic manual therapy. Only one posterior arch of the *atlas* showing the normalization of *atlas* rotation. Normalized functional spaces.

**Figure 13:**
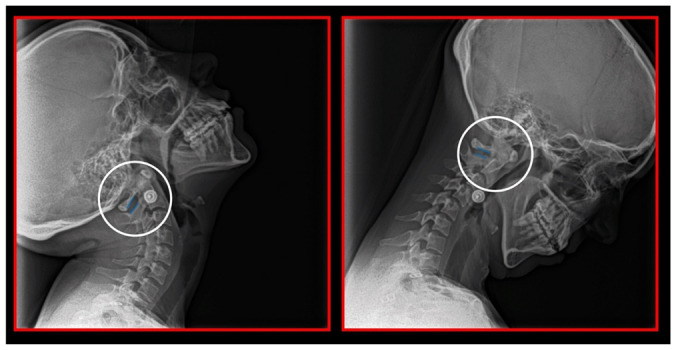
Decreased space between the posterior arch of the *atlas* and spinous process of C_2_ in maximal flexion and extension of the cervical spine. Dynamic lateral cephalometric study.

**Figure 14:**
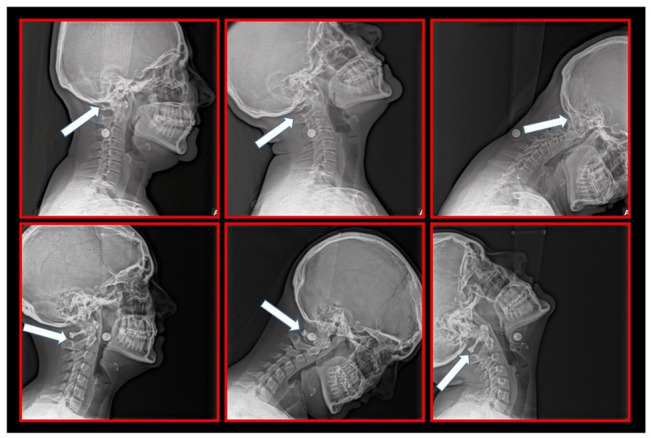
Relations among the occiput, C_1_ and C_2_ vertebrae. **Upper row:** Static *atlas* on the occiput causing mechanical compression and irritation of the C_1_ nerve root, causing occipital supraorbital pain. **Lower row:** Static *atlas* on the *axis* causing additional complaints at the level of the C_2_ nerve root, including unilateral craniofacial pain.

The gold standard and most important basic imaging method for the evaluation of COP source is neck X-ray, i.e., frontal X-ray of the upper cervical vertebrae (dorso-ventral image of head with mouth open to maximum) and lateral head and neck image. From these images, the following information can be obtained: general alignment of the cervical spine and head, presence, position (rotation, shift—spondylolisthesis), and morphology of particular vertebrae, changes suggestive of arthritis or cranio-cervical instability, the presence of ponticulus posticus, the position of the hyoid bone, jaw and teeth relationship, and other important changes (Figure 10, Figure 11, Figure 12, Figure 13 and Figure 14). In addition, particular distances and angle measurements can be easily obtained. These images can be taken by classical medical X-ray, available in most hospitals, or from cephalographs, available in orthodontic or maxillofacial surgical departments. Thus, almost every medical specialist, including a dentist, can obtain this examination quickly and cheaply.

### 10.2. Computed Tomography of Cervical Spine

**Figure 15:**
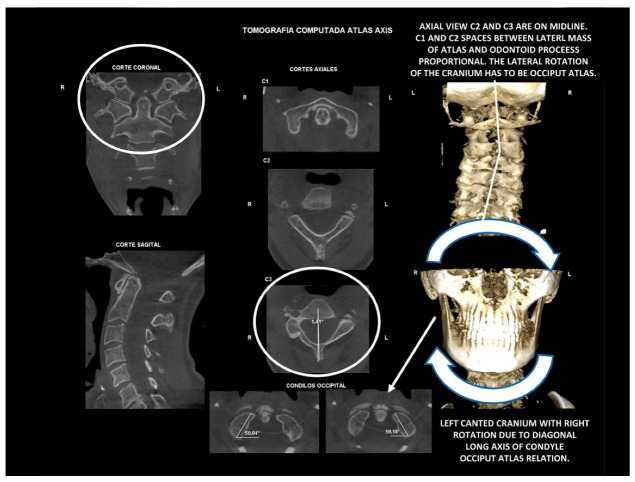
Diagnosis. *Atlas*-*axis* three-dimensional high-resolution tomography. The cranium is canted with lower cervical spine compensation. It is an occiput-*atlas* lateral or coronal plane disorder. Diagnosed as normal axial and coronal *atlas*-*axis* relation. Habitually, it is not diagnosed or diagnosed as normal craniovertebral relation.

**Figure 16:**
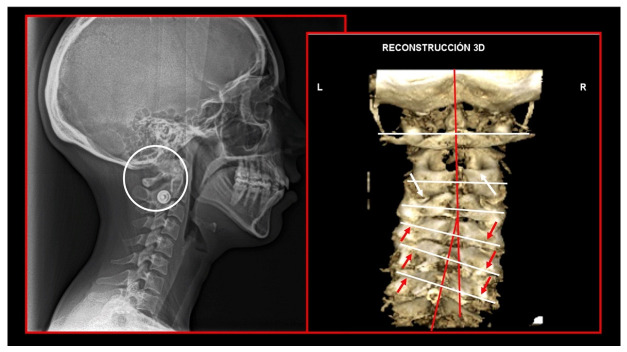
Decreased functional space between posterior arch of the *atlas* and spinous process of C2. Static *atlas* on the *axis*. Loss of cervical lordosis. Scoliosis. **Left:** lateral cephalometric radiography. **Right:** three-dimensional computed tomography reconstruction.

**Figure 17:**
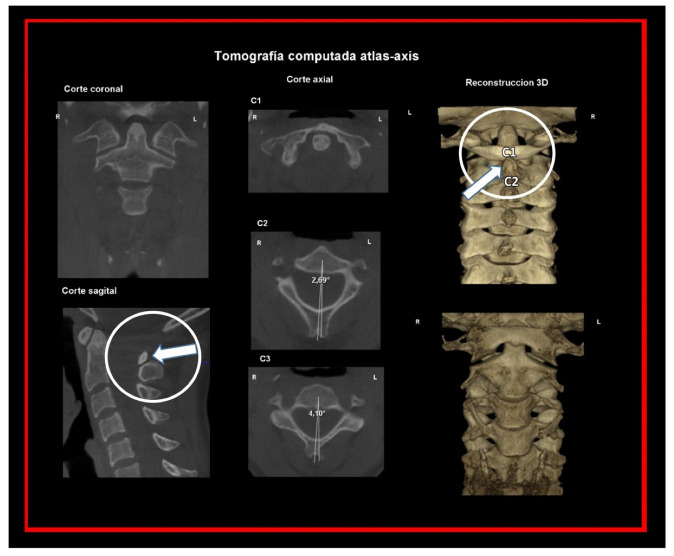
C_2_-C_3_ segment. The angle of the spinous process of the *axis* determines the degree of *axis* rotation.

CT, particularly three-dimensional (3D), high-resolution tomography, should be considered when there is a high index of suspicion for an osseous pathology and/or rotation or suspected malposition of a vertebra (Figure 15, Figure 16 and Figure 17). It should be noted that osteoarthritic changes are common with advancing age, and the presence of these findings alone on imaging is not diagnostic for COP. Barmherzig and Kingston report from their experience that over-investigating a patient with a clinical history consistent with a primary headache disorder such as migraine carries the risk of discovering these incidental changes [10]. This has the potential for harm in creating patient doubt around the initial primary headache diagnosis. Therefore, there should be a reasonable clinical suspicion of COP prior to initiating these investigations [10]. Moreover, it should be performed in case of significant intervertebral misalignment [70,71]. Although, we can perform CT with classical medical CT scan, it might be better to perform cone-beam CT (CBCT), to minimize the radiation dose [128]. Moreover, CBCT offers the acquisition of an image with high accuracy, reducing the evaluation (measurement) bias and improving the reliability, including the evaluation of data from scientific studies [50,51,129]. To date, CBCT is available in an increasing number of ambulatory offices, becoming the standard examination modality in ENT, dentistry, and cranio-maxillofacial surgery [129].

### 10.3. Magnetic Resonance Imaging of Cervical Spine

**Figure 18:**
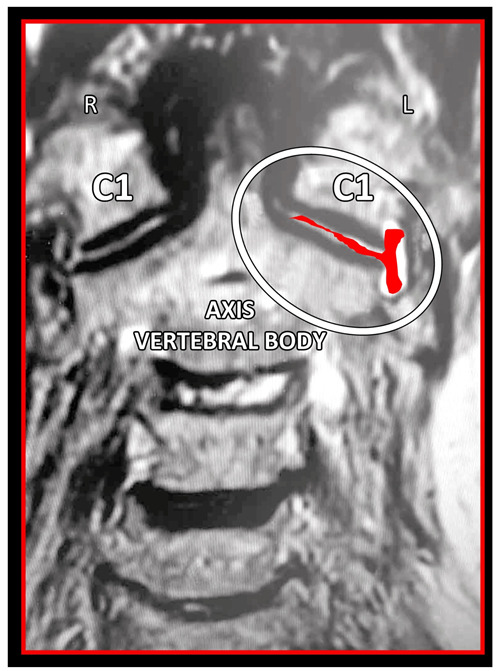
*Atlas*-*axis* synovitis. Magnetic resonance imaging showing the left lateral effusion of *atlas*-*axis* intra-joint passive congestion, with increased pain during active axial rotation of the *atlas* on *axis* to either side. C_2_ nerve root involvement with unilateral hemicranial pain.

**Figure 19:**
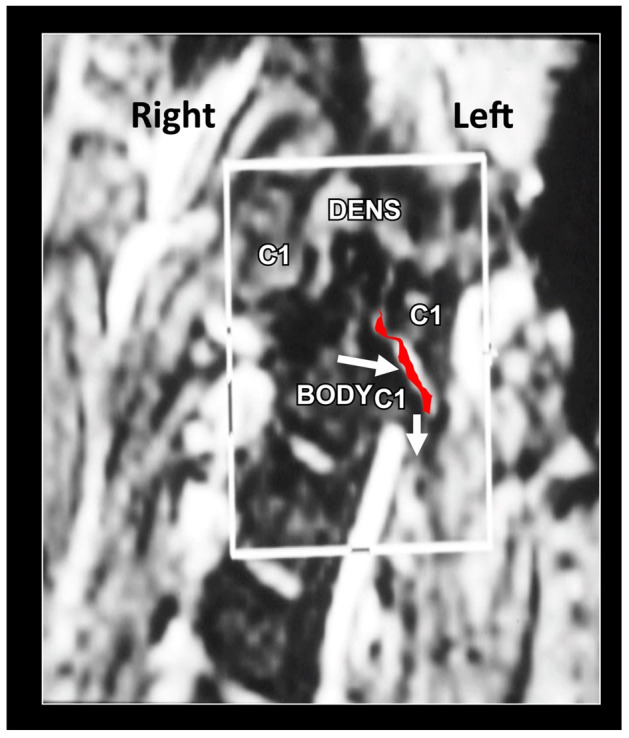
*Atlas*-*axis* gapping. Left coronal, dynamic magnetic resonance imaging (MRI), showing the lateral gapping of the left facet joint between the *atlas* and *axis*, during lateral cranial rotation (side bending) restriction. The lack of rotational component of the *axis* during lateral rotation of the cranium to the same side induces a dysfunctional pattern of cranial rotation. When the *axis* attempts to rotate towards one side, the superior lateral facet joint of *axis* drops down on the same side of the lateral rotation of the cranium, opening the gap between the lateral mass of the *axis* below the *atlas*. The lateral evaluation of the *axis* is painful on the restricted side of the lateral movement. The *axis* cannot rotate to that side and descends below the *atlas* causing capsular distension of the atlanto-axial joint. The MRI facilitates the understanding of the dysfunction to plan the treatment process and left rotation of the *axis* on the C3 vertebra. During treatment, it is important to maintain proportional rotational patterns of movement between *atlas* and *axis*.

**Figure 20:**
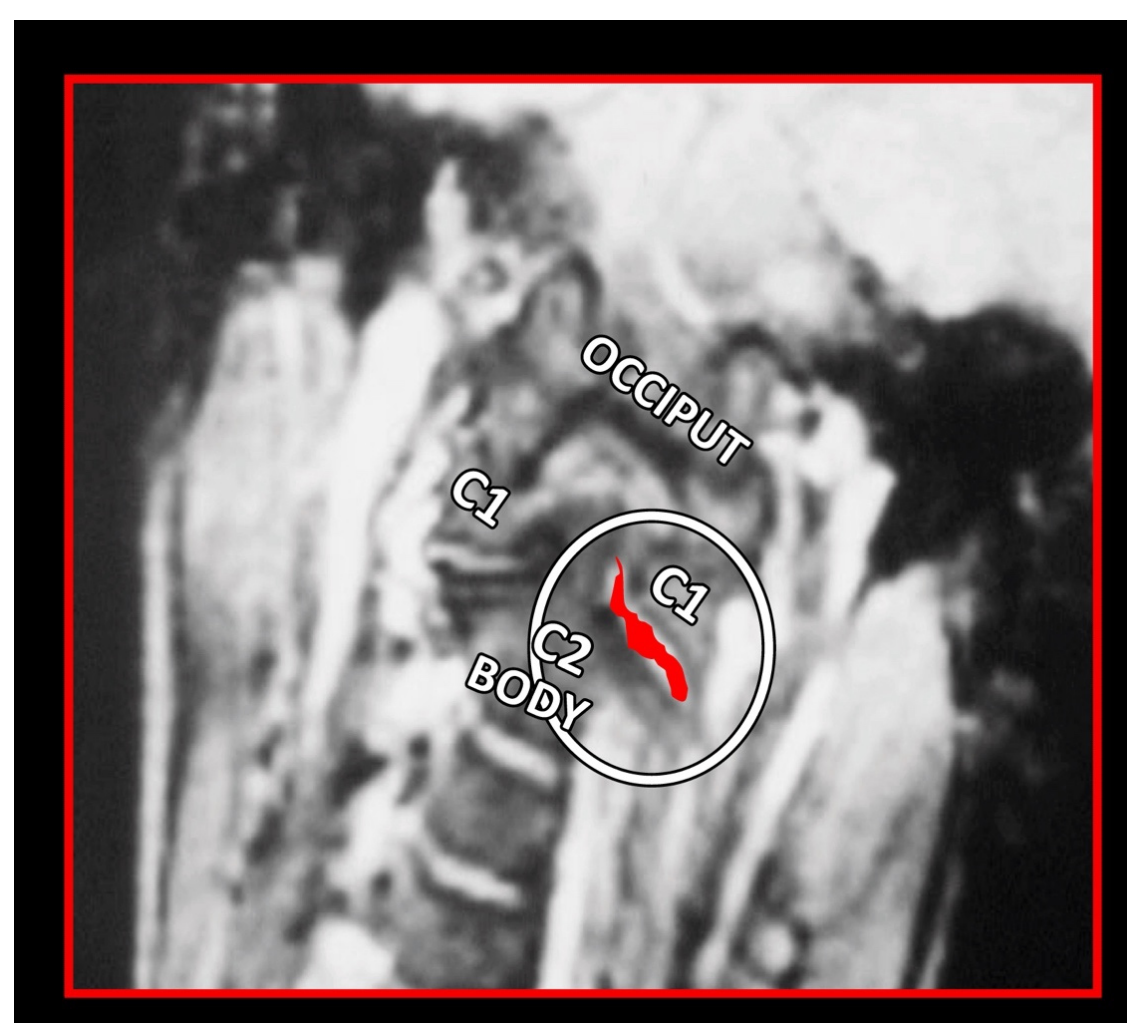
Dynamic coronal magnetic resonance imaging study. Lateral instability of *atlas*-*axis* segments. The study facilitates the understanding of the dysfunction to plan the treatment process and restore left rotation of the *axis* on the C3. Maintain proportional rotational patterns of movement between the *atlas* and *axis*.

MRI remains the imaging modality of choice, as it allows for high-quality visualization of both the cervical spine as well as the surrounding occipital and cervical soft tissues (Figure 18, Figure 19 and Figure 20). The main benefit is the zero radiation dose. Its main disadvantage is the high cost and discomfort in claustrophobic patients.

### 10.4. Sonography of Cervical Area

It may be a useful technique to evaluate the course of the occipital nerve from its origin at the C_2_ nerve root until it becomes subcutaneous at the trapezius aponeurosis. This may identify a site of entrapment in occipital neuralgia, corresponding with an enlarged, and swollen nerve appearance. The main benefit of ultrasound is its relatively low cost and zero radiation risk for the patient. However, evaluation with ultrasound relies on expertise in performing the test and interpreting results, which may render it a less valuable diagnostic modality for most clinicians [10]. Moreover, ultrasound does not provide any information about bones and joints, often necessary for the diagnosis of COP. Thus, it remains a rare modality for COP diagnosis.

## 11. Treatment

Successful treatment requires creating a therapeutic partnership with the patient, to empower the patient to take an active role in the treatment plan. Without the patient’s significant investment of time and effort, the treatment cannot be successful. Any underlying or contributory pathology should be appropriately addressed and managed. Interestingly, with the spread of COVID-19 disease during the recent pandemic, teledentistry has been introduced in the management of patients with temporomandibular disorders [130].

### 11.1. Conservative Treatment

Conservative and non-pharmacologic therapy should be preferred to pharmacologic approaches. Among these approaches, orthopedic manual therapy (targeted physiotherapy) should be the first choice. A randomized-control trial looking at spinal manipulation therapy in a cohort of highly selected patients with COP, in the absence of contraindications to spinal manipulation therapy, suggested a linear dose-response relationship between spinal manipulation therapy therapeutic sessions and days with COP, with a reduction in headache days sustained to 52 weeks after the start of therapy [131]. Another study showed that treatment with manual therapy, specific exercises, or manual therapy plus exercises was significantly more effective at reducing headache frequency and intensity than generalized care by a general practitioner [132]. Approximately 76% of patients achieved a more than 50% decrease in headache frequency and 35% achieved complete relief at the 7-week follow-up. At 12 months, 72% had a more than 50% decrease in headache frequency [132].

### 11.2. Orthopedic Manual Therapy

**Figure 21:**
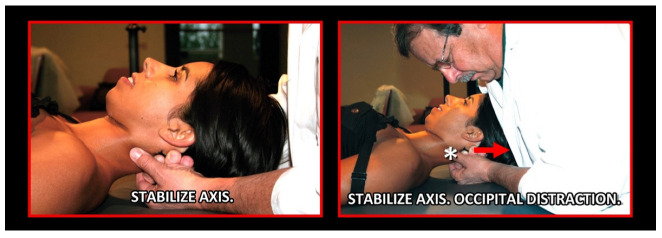
Initial orthopedic manual treatment. Source: Rocabado Institute, Chile. Long axis distraction of the cervical spine (C_0_–C_1_–C_2_).

**Figure 22:**
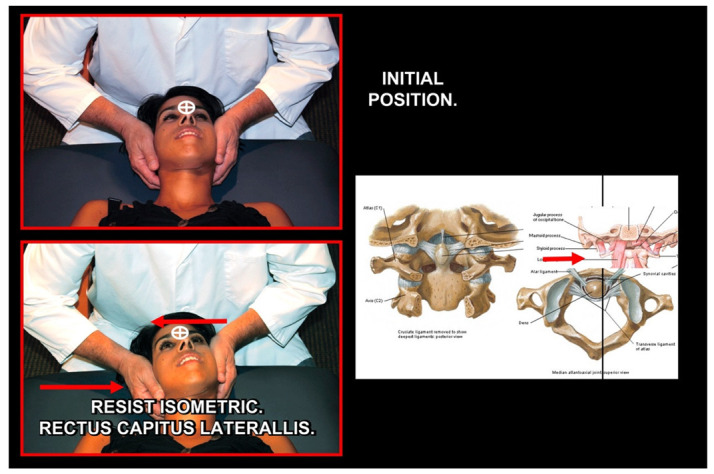
Normalization of C_2_ left rotation. Source: Rocabado Institute, Chile. Employing alar ligament and rectus capitis lateralis using neuromuscular techniques.

**Figure 23:**
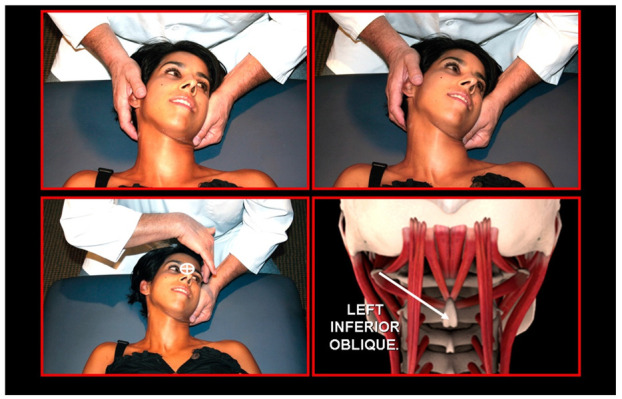
*Atlas* derotation, left direction. Source: Rocabado Institute, Chile. Employing left inferior oblique and right anterior rectus capitis muscles using neuromuscular techniques.

**Figure 24:**
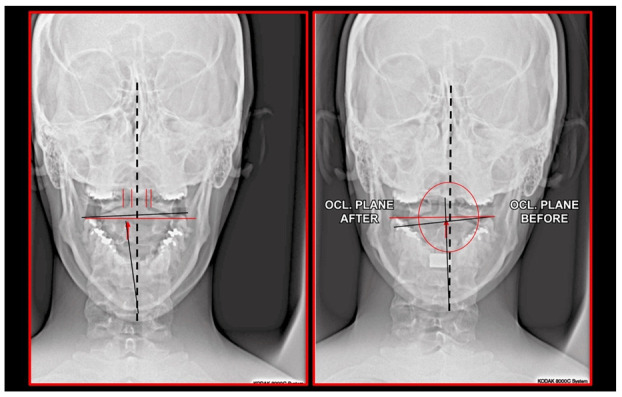
Radiographic situation before and after one session of orthopedic manual therapy.

**Figure 25:**
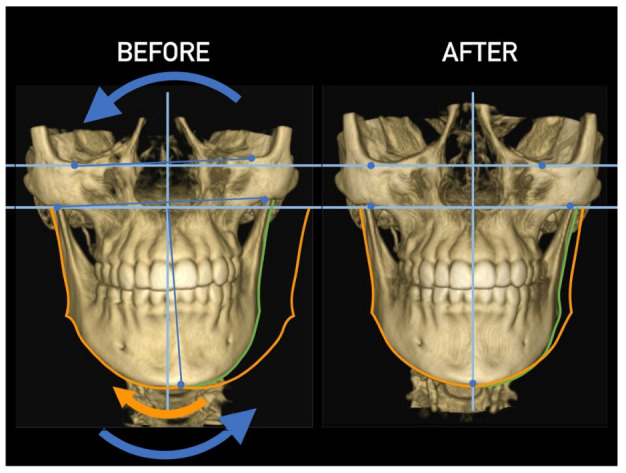
Effect of cervical vertebrae alignment. Anterior view.

**Figure 26:**
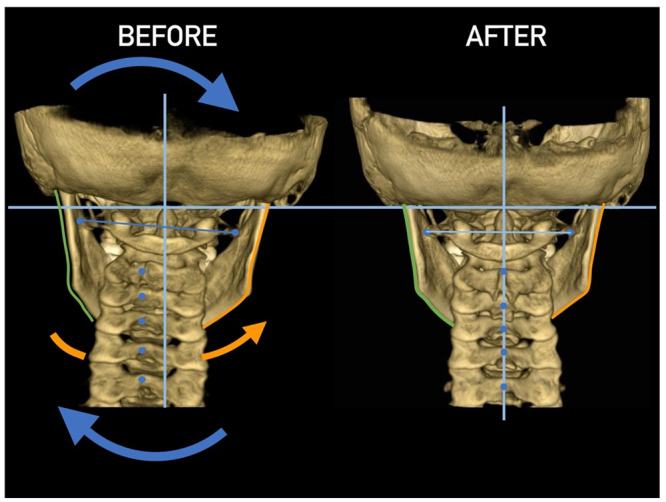
Effect of cervical vertebrae alignment. Posterior view.

**Figure 27:**
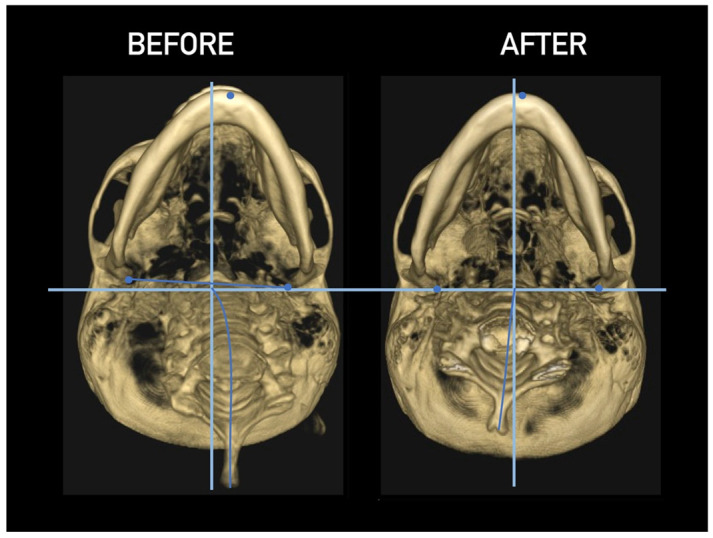
Effect of cervical vertebrae alignment. Inferior view.

**Figure 28:**
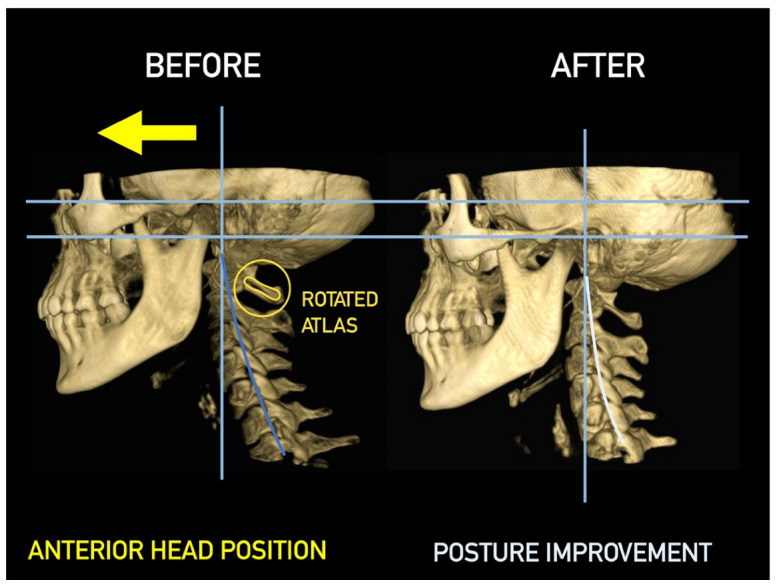
Effect of cervical vertebrae alignment. Lateral view.

Orthopedic manual therapy comprises effective differential diagnosis and treatment approach of both functional disturbances and effectiveness of motor activity in any synovial joint of the body, its connective tissue that holds the joint together and abnormal muscle function (Figure 21, Figure 22, Figure 23, Figure 24, Figure 25, Figure 26, Figure 27 and Figure 28), including the shortening, weakening, and muscle imbalance and, most important, the loss of muscle chain disconnections altering rest position. This leads to the dysfunction of time direction and load of the joint surfaces and progression to joint wear, tear, joint pain, and degenerative joint diseases. Together with a history of patient complaint in a friendly environment, a thorough and functional total body examination, and a concomitant sensitive palpatory technique with specific joint mobility of the locomotor area involved, one can utilize certain criteria that help determine if and what manipulative or total body stabilization exercise-program treatment is recommended in that specific circumstance. Criteria used for the indication of orthopedic manual therapy include localized and referred pain, observation of soft-tissue abnormalities and the zone of irritation, pathologic motion barrier, characterized by motion restriction or hypomobility, or benign joint systemic hypermobility. Muscle imbalance always presents as regional, such as muscle weakening, shortening, or general myotendinous imbalance and/or enthesitis. The first effort or provisional treatment attempt is of great concern. After the expert opinion of treatment and elimination of possible contraindications, the manual therapist will be able to make the first diagnosis and prescribe an initial approach to treatment.

### 11.3. Correction of Body Posture to Address TMJ Dysfunction

**Figure 29:**
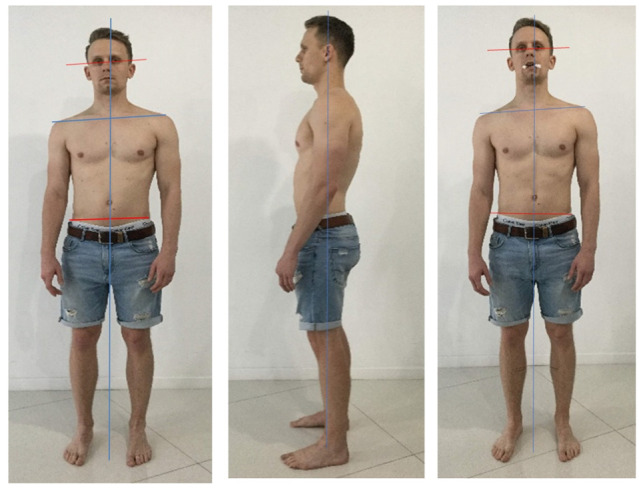
A 28-year-old patient with pain (5/10) and nonspecific muscle weakness in the right hip, evaluated by active hip flexion (pain) and unipodal balance (weakness). The informed consent from the patient was obtained for publication of photos. Source: Rocabado Institute, Chile. Note the misalignments at the shoulders (most evident), and the pelvis (less evident, however, the object of our intervention). The head is presented in the side bending/rotation to the right, and forwarded. The body posture is reassessed in both planes, this time avoiding occlusal contact with a soft disposable element. Although the shoulder misalignment is maintained, soft changes at the pelvic level can be seen: hip flexion’s pain is 1-2/10 (lower), and unipodal stability improves subjectively, although weakness persists. There are no significant changes in the sagittal plane. Such clinical findings lead us to consider the management of local conditions that are not necessarily the cause, but rather the consequence of alterations in complex kinematic chains to be considered, to increase the effectiveness of interventions, combining dentistry and physiotherapy in such clinical situation’s management.

As early as the first half of the 20th century, Thompson and Brody described the influence of posture of the body on the position of the jaw [133]. Hansson et al. and Freesmeyer suggested that an alteration in the position of the hips may be an etiological cause for cranio-mandibular dysfunctions [134,135,136]. Gelb provided an important approach to the diagnosis and treatment of cranio-mandibular dysfunctions, pointing out that alterations in posture play an etiological role in cranio-mandibular dysfunctions, and proposes that dysfunctional treatment includes the correction of body posture [137] (Figure 29). Thus, Bergbreiter found a relationship between the alterations of the posture of the hips and TMJ, finding a prevalence of joint noise in TMJ on the side of the body with a lower hip [138]. Similarly, studies by Stute showed that TMJ alterations are more frequent on the same side of the body with the lowest hip [139]. Other authors report that in patients with postural alterations, the sensitivity to palpation of the masticatory muscles is increased [140,141].

Shup and Zernial propose that the anatomical relationships explain how the postural alterations of the hips influence the position of the head [142]. These would be the relationship between the sphenobasilar (sphenooccipital) synchondrosis and sacral bone, which is made through the dura and the muscle chains made up of the masticatory, hyoid, flexor, and extensor muscles of the neck and dorsal muscles with the muscles of the hips. This finding can be explained through kinematic chains, which in the human body represent circuits in the continuity of direction and planes through which the organizing forces propagate, generating adaptive compensations based on three principles: Balance, Economy and Comfort (no pain). 

Under normal conditions, posture regulators are found in the support of the feet. These are mediated by two pairs of nerves, sensory afferents and motor efferents, and involve approximately 10% of the cerebral cortex. In conditions of dysfunction, posture mediation involves the stomatognathic system, through the information provided by TMJ structures [143] and occlusion, since the cranio-mandibular system joins the anterior and posterior kinematic chains, and, therefore, this system of complex control involves six cranial nerves and approximately 38% of the cerebral primary sensory and motor cortices. 

In an adaptive scheme, the body will attempt to maintain balance, even if the system is not economical, but the priority will always be no pain. Thus, body posture affects balance in the activation of the kinematic chains. Following the premise that “Posture control is the result of a complex system that includes different sensor and motor components from visual, somatosensory and vestibular information”, at the central level, there is a reciprocal influence between the trigeminal nerve and the vestibular nucleus, responsible for the masticatory function and balance control, respectively, and between the masticatory and cervical muscles. This influence would explain that dental malocclusions significantly impair posture control (and vice versa), even though research has not been conclusive.

Subcranial vertebral rotation conditions (Occiput-*atlas*-*axis*), generate three-dimensional positional changes at the temporomandibular articular (mandibular) fossa, as it forms an integrative part of the base of the skull, which directly affects the static and dynamic TMJ alignments, and can generate compressive and shear forces both at rest and during activity [45,143]. If we also consider the relationship between the vestibular system and balance, it is not surprising that the muscles involved in postural balance are in a condition of reflex inhibition, which may explain the changes in sensitivity and pain reported at the level of the masticatory musculature and iliac torsion phenomena (“lower hip”). It is, therefore, necessary that the study and diagnosis of cranio-mandibular dysfunctions/pathologies also consider the global analysis of the patient’s posture.

### 11.4. Treatments Based of Concepts of Trigeminal-Cervical Convergence

Treating patients with headaches and facial pain with or without synovial temporomandibular disorders and dysfunctions based on the neurological aspect has significant advantages. Upper-cervical nociceptive neurons are the point of convergence between the cervical spine, mandibular, and upper jaw nociceptive neurons. This provides a neuroanatomic interrelation between synovial TMJ and cervical spine positional faults, with or without degeneration and/or pain. This suggests that synovial TMJ disorders can often overlap with cervical-spine disorders or pathology [144]. This is crucial when formulating a diagnosis or treatment plan with a broad perspective of a holistic well-being approach.

The convergence of cervical and trigeminal afferents on second-order neurons in the trigemino-cervical nucleus may refer to pain from the upper cervical spine into the head and face. Furthermore, “bi-directional interactions” between trigeminal and upper cervical afferents may also explain neck symptoms of trigeminal origin (e.g., migraine) [145].

In orthopedic manual therapy, the musculoskeletal concept is that the synovial joints, soft tissues that hold the joint together, and the muscles that move the joint have the same innervation; however, the nerves that supply the joint also supply the muscles that move the joint. This fundamental principle is also known as Hilton’s law [20,146]. When the intra-joint terminals become irritated, the muscles that move the joint experience reflex muscle contraction. This increases the intra-joint tension and a vicious cycle is set up. Reinforcing this physiological concept is the fact that soft tissue damage precedes hard tissue injuries.

Details of the Rocabado principles of conservative temporomandibular joint dysfunction (TMD) treatment are beyond the scope of this article, but are described in detail in our previous studies [16,70,71,147]. Briefly, the TMD orthopedic manual therapy consists of the use of occlusal 24/7 splint, distraction of TMJ in order to increase the joint space and the vertical capsular/condylar dimension and wide range of myofascial techniques at the level of masticatory musculature, supra- and infrahyoid muscles, suboccipital, and paravertebral muscles [16,70,71]. Once the goal of cranio-cervico-mandibular and occlusion stability is achieved, the patient continues with a total general musculoskeletal stabilization program with a long-term approach and dental and physiotherapy control every six months [147]. Thus, the musculo-fascio-ligamento-capsular aspects of TMD, and not only the temporomandibular joint itself, is considered in both diagnosis and treatment. Importantly, both TMD and neck pain could cause facial pain, and are frequently associated with the development of craniofacial allodynia during painful exacerbation. The central sensitization that could be developed in these patients needs to be taken into account, as well [148,149].

### 11.5. Axis and Atlas Derotation

**Figure 30:**
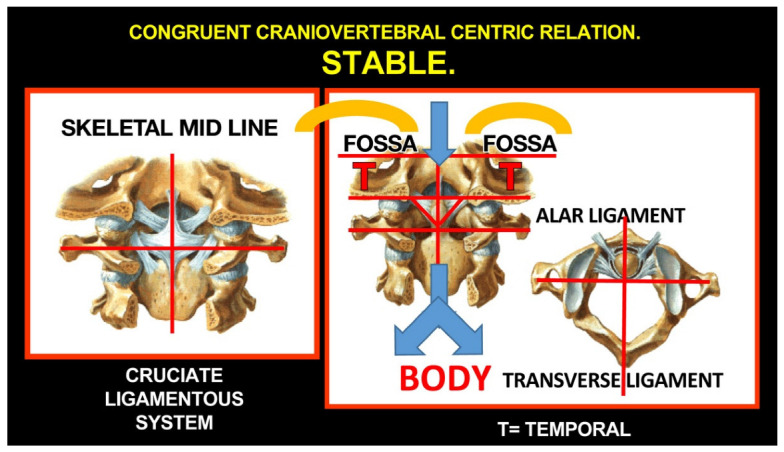
Stable, centric relation of the craniovertebral joints.

Considering these strong concepts in treating a facial pain or headache of cervical origin with or without concomitant synovial TMJ disorder, the position of the head in space in three dimensions, sagittal, coronal, and axial relation to the rest of the body, this concept becomes fundamental. It determines the functional position of the occipital bone with the *atlas* (first cervical vertebra) supporting through the condyles of the occiput, and the weight of the head. The *atlas* needs to be horizontal over the segment of C_2_–C_3_, with the *axis* (second cervical vertebra) in the skeletal midline to distribute the total weight of the head to the rest of the body (Figure 30). This is a stable, centric relation of the craniovertebral joints. The craniovertebral centric relation determines a horizontal transverse occlusal plane of the upper maxilla and mandible.

Importantly, the harmony of osseous structures remains the reflection of the dynamics of soft-tissue biomechanics. Moreover, soft-tissue damage proceeds in hard-tissue injury of the bone and joints. Cranial position and movement are controlled by more than 20 pairs of cervical muscles. This enormous dynamic condition controls not only the cranium, but determines the position of the mandibular fossa of the temporal bone in the three planes of space and undoubtfully the 3D position of the maxilla. Once we normalize the occipito-atlanto-axial relationship (not earlier!), we can begin determining the mandibulo-maxillary relations, or condyle-fossa congruent position with or without the disc interposed, or how mandibular occlusal contacts relate to maxillary occlusal contacts for rest position. These 40 muscles work simultaneously to stabilize and control the cranium as a stable foundation for mandibular proportional patterns of movement, which, consequently, can affect the intra-joint condyle-disc/disc-temporal arthrokinematics.

The cranium through the cervical spine in lordosis (physiological curvature of the cervical spine) needs to be stabilized over the horizontal shoulder girdle to permit the coordinated action of the masticatory, supra- and infra-mandibular, prevertebral, as well as middle and inferior pharyngeal-constrictor muscles with their antagonistic, deep craniovertebral and superficial paravertebral cervical muscles. Particular orthopedic manual therapy of the most common positional fault syndromes considers that these biomechanical conditions are illustrated, showing they are not problems associated with age, as often stated.

### 11.6. Treatments Focused on Temporomandibular Joint

Once the optimal craniovertebral position is acquired and stabilized and, thereby, the optimal position of mandibular condyle-fossa relationship is obtained, initial (“default”) conditions for the treatment of the TMJ are obtained [45]. If the occlusion does not change significantly and the mandible can move freely in all directions without pain, an occlusal splint is not necessary. However, this happens in few cases. In most cases, an occlusal splint is necessary to provide free movement of the mandible in all directions, at the beginning of treatment in the anteroposterior direction (protrusion/retrusion of mandible) in particular. A plastic splint is most often fabricated on the upper jaw, so its bottom part is rigid and flat, with only slightly increased canine regions to obtain a basic, mutually protected occlusion [26,150,151]. In subsequent orthopedic manual therapy visits, the splint is grinded to obtain at least a 16-point contact with the lower teeth at rest, without right-left imbalance.

During these visits, the manual therapist works with TMJ using particular techniques, including long axis distraction of the joint, mobilization therapy, and a wide range of soft techniques affecting the masticatory (and other) muscles and craniomandibular ligaments. Further, help from a specialist in maxillofacial surgery may rarely be necessary; however, such treatment should be planned (its necessity diagnosed) and discussed with the patient prior to the start of the treatment.

### 11.7. Treatment of Occlusion

When the optimal occlusion is obtained, aligning the teeth to obtain an optimal maxillo-mandibular tooth relationship should follow, including proper contact of all upper and lower teeth according to the tripod concept and functional anterior guidance (separation of the lateral teeth when the mandible is protruded; sagittal Christensen’s phenomenon), as well as lateral guidance (separation of the contralateral lateral teeth when the mandible is in laterotrusion; transversal Christensen’s phenomenon), led by the upper canine (canine guidance) or more teeth (group function/group guidance). The most important factors in occlusion remain the absence of traumatic articulation, artificial mandibular guidance and/or occlusal interferences, in particular, mediotrusive molar interferences, thus avoiding muscular avoidance patterns towards these occlusal “hot spots” and other consequent problems [26] (for details, see Dawson, 2007; Greven et al., 2020 [152,153]). The occlusal alignment can be obtained by orthodontic, restorative, prosthetic dentistry, or their combination. Such alignment of the teeth allows the dental specialist to eliminate the splint and obtain optimal functional (including masticatory) ability of the dentition, without pain or disturbances.

### 11.8. Other Therapeutic Modalities

Apart from the principal approaches, there are several others, which may have adjuvant (not primarily curative) potential. Anesthetic nerve blocks generally play a dual role in both supporting diagnosis and pain relief. However, in the case of occipital neuralgia or COP, the response to anesthetic nerve blocks should not be considered pathognomonic, as its specificity is poor, and other primary and secondary headache disorders may also respond [10].

Transcutaneous electrical nerve stimulation therapy has been used in the conservative management of cervicogenic headaches, with reported benefits [154,155]. However, given the inherent difficulty of realistic placebo and blinding in TENS studies, the results should be interpreted cautiously [156].

Acupuncture or other alternative methods may be beneficial. 

Psychotherapy, including meditation (e.g., mindfulness) and cognitive-behavioral therapy remains important supplemental therapies in a significant number of patients, due to the beneficial effect as they undergo this often long and demanding treatment process. Although most psychological problems are secondary, it still has an important therapeutic potential.

Pharmacological and surgical interventions should be reserved for selected patient populations in whom all other conservative and minimally invasive options have failed, to be weighed against the potential risk. They are not the primary focus of this article; thus, they are reviewed elsewhere [10].

## 12. Conclusions

The etiopathogenesis of COP is complex, but the data are available, understandable, and most importantly, clinically applicable. The key is the understanding of the mechanism of trigeminocervical functional convergence as well as the tricentric concept. Based on the understanding of neuroanatomical and neurophysical, neuromuscular relations, a precise diagnosis and a successful conservative treatment of these patients, based mainly on orthopedic manual therapy and occlusal treatment, is possible, reliable and reproducible.

## Figures and Tables

**Table 1:** The International Classification of Headache Disorders (ICHD-3) diagnostic criteria for cervicogenic headache according to the Headache Classification Committee of the International Headache Society (2018).

A. Any headache fulfilling criterion C.
B. Clinical and/or imaging evidence of a disorder or lesion within the cervical spine or soft tissues of the neck, known to be able to cause headache.
C. Evidence of causation demonstrated by at least two of the following: 1. headache has developed in temporal relation to the onset of the cervical disorder or appearance of the lesion; 2. headache has significantly improved or resolved in parallel with improvement in or resolution of the cervical disorder or lesion; 3. cervical range of motion is reduced and headache is made significantly worse by provocative maneuvers; 4. headache is abolished following diagnostic blockade of a cervical structure or its nerve supply.
D. Not better accounted for by another ICHD-3 diagnosis.

## Data Availability

Not applicable.

## References

[1] Verma S., Tripathi M., Chandra P.S. (2021). Cervicogenic Headache: Current Perspectives. Neurol. India..

[2] Van Suijlekom H.A., Lamé I., Stomp-van den Berg S.G., Kessels A.G., Weber W.E. (2003). Quality of Life of Patients with Cervicogenic Headache: A Comparison with Control Subjects and Patients With Migraine or Tension-Type Headache. Headache.

[3] Fernandez M., Moore C., Tan J., Lian D., Nguyen J., Bacon A., Christie B., Shen I., Waldie T., Simonet D. (2020). Spinal manipulation for the management of cervicogenic headache: A systematic review and meta-analysis. Eur. J. Pain..

[4] Bogduk N. (1992). The anatomical basis for cervicogenic headache. J. Manipulative Physiol. Ther..

[5] Bogduk N., Govind J. (2009). Cervicogenic headache: An assessment of the evidence on clinical diagnosis, invasive tests, and treatment. Lancet Neurol..

[6] Bogduk N. (2014). The neck and headaches. Neurol. Clin..

[7] Headache Classification Subcommittee of the International Headache Society (2004). The International Classification of Headache Disorders: 2nd edition. Cepthalagia.

[8] Headache Classification Subcommittee of the International Headache Society (2013). The International Classification of Headache Disorders 3rd edition (beta version). Cephalgia.

[9] Sjaastad O., Fredriksen T.A., Pfaffenrath V. (1998). Cervicogenic Headache: Diagnostic Criteria. Headache. J. Head Face Pain.

[10] Barmherzig R., Kingston W. (2019). Occipital Neuralgia and Cervicogenic Headache: Diagnosis and Management. Curr. Neurol. Neurosci. Rep..

[11] Fredriksen T.A., Antonaci F., Sjaastad O. (2015). Cervicogenic headache: Too important to be left undiagnosed. J. Headache Pain.

[12] Sjaastad O., Bakkteig L.S. (2008). Prevalence of cervicogenic headache: Vaga study of headach epidemiology. Acta Neurol. Scand..

[13] Knackstedt H., Bansevicius D., Aaseth K., Grande R.B., Lundqvist C., Russel M.B. (2010). Cervicogenic headache in the general population: The Akershus study of chronic headache. Cephalalgia.

[14] Evers S. (2008). Comparison of cervicogenic headache with migraine. Cephalalgia.

[15] Lord S., Barnsley L., Wallis B., Bogduk N. (1994). Third occipital headache: A prevalence study. J. Neurol. Neurosurg. Psychiatr..

[16] Rocabado M., Iglarsh Z.A. (1991). Musculoskeletal Approach to Maxillofacial Pain.

[17] Dvorak J., Penning L., Hayek J., Panjabi M.M., Grob D., Zehnder R. (1988). Functional diagnostics of the cervical spine using computer tomography. Neuroradiology.

[18] Williams P.L., Bannister H. (1995). Gray’s Anatomy.

[19] Kikuta S., Jenkins S., Kusukawa J., Iwanaga J., Loukas M., Tubbs R.S. (2019). Ansa cervicalis: A comprehensive review of its anatomy, variations, pathology, and surgical applications. Anat. Cell Biol..

[20] Moore K.L., Dalley A.F., Agur A.M.R. (2014). Moore Clinically Oriented Anatomy.

[21] Renton T., Egbuniwe O. (2015). Pain. Part 2A: Trigeminal Anatomy Related to Pain. Dent. Update.

[22] Lazarov N.E. (2007). Neurobiology of orofacial proprioception. Brain Res. Rev..

[23] Bogduk N. (2001). Cervicogenic headache: Anatomic basis and pathophysiologic mechanisms. Curr. Pain Head Rep..

[24] Biondi D.M. (2005). Cervicogenic headache: A review of diagnostic and treatment strategies. J. Am. Osteopath. Assoc..

[25] Biondi D.M. (2005). Noninvasive treatments for headache. Expert. Rev. Neurother..

[26] Okeson J.P. (2020). Management of Temporomandibular Disorders and Occlusion.

[27] Kerr F.W. (1962). Facial, vagal and glossopharyngeal nerves in the cat. Afferent connections. Arch. Neurol..

[28] Kerr F.W. (1963). The divisional organization of afferent fibres of the trigeminal nerve. Brain.

[29] Cetas J.S., Saedi T., Burchiel K.J. (2008). Destructive procedures for the treatment of nonmalignant pain: A structured literature review. J. Neurosurg..

[30] Giovanni A., Giorgia A. (2021). The neurophysiological basis of bruxism. Heliyon.

[31] Louvi A., Yoshida M., Grove E.A. (2007). The derivatives of the Wnt3a lineage in the central nervous system. J. Comp. Neurol..

[32] Lund J.P. (1991). Mastication and its control by the brain stem. Crit. Rev. Oral Biol. Med..

[33] Cody F.W., Lee R.W., Taylor A. (1972). A functional analysis of the components of the mesencephalic nucleus of the fifth nerve in the cat. J. Physiol..

[34] Daunicht W.J., Jaworski E., Eckmiller R. (1985). Afferent innervation of extraocular muscles in the rat studied by retrograde and anterograde horseradish peroxidase transport. Neurosci. Lett..

[35] Porter J.D., Spencer R.F. (1982). Localization of morphology of cat extraocular muscle afferent neurons identified by retrograde transport of horseradish peroxidase. J. Comp. Neurol..

[36] Dessem D., Luo P. (1999). Jaw–muscle spindle afferent feedback to the cervical spinal cord in the rat. Exp. Brain Res..

[37] Goodwin G.M., Luschei E.S. (1974). Effects of destroying spindle afferents from jaw muscles on mastication in monkeys. J. Neurophysiol..

[38] Ongerboer de Visser B.W. (1982). Afferent limb of the human jaw reflex: Electrophysiologic and anatomic study. Neurology.

[39] Luschei E.S. (1987). Central projections of the mesencephalic nucleus of the fifth nerve: An autoradiographic study. J. Comp. Neurol..

[40] Daunton N.G. (1977). Sensory components of bite–force response in the rat. J. Comp. Physiol. Psychol..

[41] Eichner K. (1955). Über eine Gruppeneinteilung der Lückengebisse für die Prothetik. Dtsch. Zahnärztl. Z..

[42] Malet J. (2012). Implant Dentistry at Glance.

[43] Cheynet F., Guyot L., Richard O., Layoun W., Gola R. (2003). Discomallear and malleomandibular ligaments: Anatomical study and clinical applications. Surg. Radiol. Anat..

[44] Connelly S.T., Tartaglia G.M., Silva R.G. (2019). Contemporary Management of Temporomandibular Disorders. Fundamentals and Pathways to Diagnosis.

[45] Olmos S.R., Kritz–Silverstein D., Halligan W., Silverstein S.T. (2005). The effect of condyle fossa relationships on head posture. J. Craniomand. Pract..

[46] Von Piekartz H.J.M., Schouten S., Aufdemkampe G. (2007). Neurodynamic responses in children with migraine or cervicogenic headache versus a control group. A comparative study. Man. Ther..

[47] Wei W. (2018). Neural Mechanisms of Motion Processing in the Mammalian Retina. Annu. Rev. Vis. Sci..

[48] D’Attilio M., Filippi M.R., Femminella B., Festa F., Tecco S. (2005). The influence of an experimentally–induced malocclusion on vertebral alignment in rats: A controlled pilot study. Cranio.

[49] D’Attilio M., Scarano A., Quaranta A., Festa F., Caputi S., Piattelli A. (2007). Modification of condyle anatomy following a monolateral bite rise: A histological study in rat. Int. J. Immunopathol. Pharmacol..

[50] Cardinal L., da Silva T.R., Andujar A.L.F., Gribel B.F., Dominguez G.C., Janakiraman N. (2022). Evaluation of the three-dimensional (3D) position of cervical vertebrae in individuals with unilateral posterior crossbite. Clin. Oral Investig..

[51] Šedý J. (2021). Response to: Cardinal L, da Silva TR, Andujar ALF, Gribel BF, Dominguez GC, Janakiraman N. Evaluation of the three-dimensional (3D) position of cervical vertebrae in individuals with unilateral posterior crossbite. Clin. Oral Invest..

[52] Di Vece L., Faleri G., Picciotti M., Guido L., Giorgetti R. (2010). Does a transverse maxillary deficit affect the cervical vertebrae? A pilot study. Am. J. Orthod. Dentofacial. Orthop..

[53] McGuinness N.J., McDonald J.P. (2006). Changes in natural head position observed immediately and one year after rapid maxillary expansion. Eur. J. Orthod..

[54] Greenbaum T., Dvir Z., Reiter S., Winocur E. (2017). Cervical flexion-rotation test and physiological range of motion—A comparative study of patients with myogenic temporomandibular disorder versus healthy subjects. Musculoskelet. Sci. Pract..

[55] Korbmacher H., Koch L., Eggers-Stroeder G., Kahl-Nieke B. (2007). Associations between orthopaedic disturbances and unilateral crossbite in children with asymmetry of the upper cervical spine. Eur. J. Orthod..

[56] Milidonis M.K., Kraus S.L., Segal R.L., Widmer C.G. (1993). Genioglossi muscle activity in response to changes in anterior/neutral head posture. Am. J. Orthod. Dentofacial. Orthop..

[57] Mohl N. (1977). Head posture and its role in occlusion. Int. J. Orthod..

[58] Ohmure H., Miyawaki S., Nagata J., Ikeda K., Yamasaki K., Al–Kalaly A. (2008). Influence of forward head posture on condylar position. J. Oral Rehabil..

[59] Paco M., Duarte J.A., Pinho T. (2021). Orthodontic Treatment and Craniocervical Posture in Patients with Temporomandibular Disorders: An Observational Study. Int. J. Environ. Res. Public Health.

[60] Sandoval C., Díaz A., Manríquez G. (2019). Relationship between craniocervical posture and skeletal class: A statistical multivariate approach for studying Class II and Class III malocclusions. Cranio.

[61] Proffit W.R. (2019). Contemporary Orthodontics.

[62] McNamara J.A. (2002). Early intervention in the transverse dimension: Is it worth the effort?. Am. J. Orthod. Dentofacial. Orthop..

[63] Michelotti A., Iodice G., Piergentili M., Farella M., Martina R. (2016). Incidence of temporomandibular joint clicking in adolescents with and without unilateral posterior cross-bite: A 10-year follow-up study. J. Oral Rehabil..

[64] Fiorrilo L. (2020). Spine and TMJ: A Pathophysiology report. J. Funct. Morphol. Kinesiol..

[65] Cruccu G., Ongerboer de Visser B.W. (1999). The jaw reflexes. The International Federation of Clinical Neurophysiology. Electroencephalogr. Clin. Neurophysiol..

[66] Morquette P., Lavoie R., Fhima M.D., Lamoureux X., Verdier D., Kolta A. (2012). Generation of the masticatory central pattern and its modulation by sensory feedback. Prog. Neurobiol..

[67] Dellow P.G., Lund J.P. (1971). Evidence for central timing of rhythmical mastication. J. Physiol..

[68] Sessle B.J., Yao D., Nishiura H., Yoshino K., Lee J.C., Martin R.E., Murray G.M. (2005). Properties and plasticity of the primate somatosensory and motor cortex related to orofacial sensorimotor function. Clin. Exp. Pharmacol. Physiol..

[69] Hamm T.M., Trank T.V., Turkin V.V. (1999). Correlations between neurograms and locomotor drive potentials in motoneurons during fictive locomotion: Implications for the organization of locomotor commands. Prog. Brain Res..

[70] Rocabado M. (2018). Theoretical and Hans–on Master Class: Cervical and Craniomandibular Dysfunctions.

[71] Rocabado M. (2018). Theoretical and Hans–on Master Class II: Cervical and Craniomandibular Dysfunctions.

[72] Goadsby P.J., Ratsch T. (2008). On the functional neuroanatomy of neck pain. Cephalalgia.

[73] Campbell D.G., Parsons C.M. (1944). Referred head pain and its concomitants. J. Nerv. Ment. Dis..

[74] Feinstein B., Langton J.B.K., Jameson R.M., Schiller F. (1954). Experiments on referred pain from deep somatic tissues. J. Bone Joint Surg..

[75] Dreyfuss P., Michaelsen M., Fletcher D. (1994). Atlanto-occipital and lateral atlanto-axial joint pain patterns. Spine.

[76] Dwyer A., Aprill C., Bogduk N. (1990). Cervical zygapophysial joint pain patterns I: A study in normal volunteers. Spine.

[77] Schellhas K.P., Smith M.D., Gundry C.R., Pollei S.R. (1996). Cervical discogenic pain: Prospective correlation of magnetic resonance imaging and discography in asymptomatic subjects and pain suff erers. Spine.

[78] Grubb S.A., Kelly C.K. (2000). Cervical discography: Clinical implications from 12 years of experience. Spine.

[79] Ashina S., Bendtsen L., Lyngberg A.C., Lipton R.B., Hajiyeva N., Jensen R. (2015). Prevalence of neck pain in migraine and tension-type headache: A population study. Cephalalgia.

[80] Johnston M.M., Jordan S.E., Charles A.C. (2013). Pain referral patterns of the C1 to C3 nerves: Implications for headache disorders. Ann. Neurol..

[81] Shimohata K., Hasegawa K., Onodera O., Nishizawa M., Shimohata T. (2017). The clinical features, risk factors, and surgical treatment of cervicogenic headache in patients with cervical spine disorders. Headache.

[82] Amevo B., Aprill C., Bogduk N. (1992). Abnormal instantaneous axes of rotation in patients with neck pain. Spine.

[83] Watson D.H., Trott P.H. (1993). Cervical headache: An investigation of natural head posture and upper cervical flexor muscle performance. Cephalalgia.

[84] Okeson J.P. (1996). Orofacial Pain: Guidelines for Assessment, Diagnosis and Management.

[85] Paesani D.A. (2010). Bruxism: Theory and Practice.

[86] Harness D.M., Peltier B. (1992). Comparison of MMPI scores with self-report of sleep disturbance and bruxism in the facial pain population. Cranio.

[87] Pierce C.J., Chrisman K., Bennett M.E., Close J.M. (1995). Stress, anticipatory stress, and psychologic measures related to sleep bruxism. J. Orofac. Pain.

[88] Rugh J.D., Harlan J. (1988). Nocturnal bruxism and temporomandibular disorders. Adv. Neurol..

[89] Bandodkar S., Tripathi S., Chand P., Singh S.V., Arya D., Kumar L., Singh M., Singhal R., Tripathi A. (2022). A study to evaluate psychological and occlusal parameters in bruxism. J. Oral Biol. Craniofac. Res..

[90] Franks A.S. (1968). Cervical spondylosis presenting as the facial pain of temporomandibular joint disorder. Ann. Phys. Med..

[91] Koopman J.S., Dieleman J.P., Huygen F.J., de Mos M., Martin C.G., Sturkenboom M.C. (2009). Incidence of facial pain in the general population. Pain.

[92] Choi I.I., Jeon S.R. (2016). Neuralgias of the head: Occipital neuralgia. J. Korean Med. Sci..

[93] Khanfour A.A., El Sekily N.M. (2015). Relation of the vertebral artery segment from C1 to C2 vertebrae: An anatomical study. Alexandria J. Med..

[94] Allen W. (1879). The varieties of the atlas in the human subject, and the homologies of its transverse processes. J. Anat. Physiol..

[95] Pekala P.A., Henry B.M., Pekala J.R., Hsieh W.C., Vikse J., Sanna B., Walocha J.A., Tubbs R.S., Tomaszewski K.A. (2017). Prevalence of foramen arcuale and its clinical significance: A meta-analysis of 55,985 subjects. J. Neurosurg. Spine.

[96] Friedrich R.E. (2014). Ponticulus posticus is a frequent radiographic finding on lateral cephalograms in nevoid basal cell carcinoma syndrome (Gorlin-Goltz syndrome). Anticancer Res..

[97] Limousin C.A. (1980). Foramen arcuale and syndrome of Barre-Lieou. Its surgical treatment. Int. Orthop..

[98] Li Y., Peng B. (2015). Pathogenesis, Diagnosis, and Treatment of Cervical Vertigo. Pain Physician.

[99] Travell J.G., Simons D. (2013). Myofascial Pain and Dysfunction.

[100] Zhuang X., Tan S., Huang Q. (2014). Understanding of myofascial trigger points. Chin. Med. J..

[101] Gerwin R.D., Dommerholt J., Shah J.P. (2004). An expansion of Simons integrated hypothesis of trigger point formation. Curr. Pain Headache Rep..

[102] Sharav Y., Singer E., Schmidt E., Dionne R.A., Dubner R. (1987). The analgesic effect of amitriptyline on chronic facial pain. Pain.

[103] Clarkson E., Jung E. (2020). Atypical Facial Pain. Dent. Clin. North Am..

[104] May A., Hoffmann J. (2021). Facial pain beyond trigeminal neuralgia. Curr. Opin. Neurol..

[105] Marklund M., Braem M.J.A., Verbraecken J. (2019). Update on oral appliance therapy. Eur. Respir. Rev..

[106] Antonaci F., Ghirmai S., Bono S., Sandrini G., Nappi G. (2001). Cervicogenic headache: Evaluation of the original diagnostic criteria. Cephalalgia.

[107] Van Suijlekom J.A., de Vet H.C.W., van den Berg S.G.M., Weber W.E.J. (1999). Interobserver reliability of diagnostic criteria for cervicogenic headache. Cephalalgia.

[108] van Suijlekom H.A., de Vet H.C.W., van den Berg S.G.M., Weber W.E.J. (2000). Interobserver reliability in physical examination of the cervical spine in patients with headache. Headache.

[109] Grzesiak R.C. (1991). Psychologic considerations in temporomandibular dysfunction. A biopsychosocial view of symptom formation. Dent. Clin. North Am..

[110] Fillingim R.B., Ohrbach R., Greenspan J.D., Sanders A.E., Rathnayaka N., Maixner W., Slade G.D. (2020). Associations of Psychologic Factors with Multiple Chronic Overlapping Pain Conditions. J. Oral Facial Pain Headache.

[111] Van Suijlekom H., Van Zundert J., Narouze S., Van Kleef M., Mekhail N. (2010). Cervicogenic headache. Pain Pract..

[112] Lampl C., Rudolph M., Deligianni C.I., Mitsikostas D.D. (2015). Neck pain in episodic migraine: Premonitory symptom or part of the attack?. J. Headache Pain.

[113] Kuhn W.F., Kuhn S.C., Gilberstadt H. (1997). Occipital neuralgias: Clinical recognition of a complicated headache. A case series and literature review. J. Orofac. Pain.

[114] de Sousa J.E., Halfon M.J., Bonardo P., Reisin R.C., Fernández Pardal M.M. (2005). Different pain patterns in patients with vertebral artery dissections. Neurology.

[115] Saeed A.B., Shuaib A., Al Sulaiti G., Emery D. (2000). Vertebral artery dissection: Warning symptoms, clinical features and prognosis in 26 patients. Can. J. Neurol. Sci..

[116] Campos C.R., Calderaro M., Scaff M., Conforto A.B. (2007). Primary headaches and painful spontaneous cervical artery dissection. J. Headache Pain.

[117] Hack G.D., Koritzer R.T., Robinson W.L., Hallgren R.C., Greenman P.E. (1995). Anatomic relation between the rectus capitis posterior minor muscle and the dura mater. Spine.

[118] Hallgren R.C., Pierce S.J., Prokop L.L., Rowan J.J., Angela L.S. (2014). Electromyographic activity of rectus capitis posterior minor muscles associated with voluntary retraction of the head. Spine J..

[119] Hallgren R.C., Pierce S.J., Sharma D.B., Rowan J.J. (2017). Forward Head Posture and Activation of Rectus Capitis Posterior Muscles. J. Am. Osteopath. Assoc..

[120] Blumenfeld A., Siavoshi S. (2008). The challenges of cervicogenic headache. Curr. Pain Headache Rep..

[121] Lance J.W., Anthony M. (1980). Neck tongue syndrome on sudden turning of the head. J. Neurol. Neurosurg. Psychiatr..

[122] Bogduk N. (1981). An anatomical basis for neck tongue syndrome. J. Neurol. Neurosurg. Psychiatr..

[123] Jansen J., Markakis E., Rama B., Hildebrandt J. (1989). Hemicranial attacks or permanent hemicrania—A sequel of upper cervical root compression. Cephalalgia.

[124] Poletti C.E., Sweet W.H. (1990). Entrapment of the C2 root and ganglion by the atlanto-epistrophic ligament: Clinical syndrome and surgical anatomy. Neurosurgery.

[125] Jansen J., Bardosi A., Hildebrandt J., Lucke A. (1989). Cervicogenic, hemicranial attacks associated with vascular irritation or compression of the cervical nerve root C2. Clinical manifestations and morphological findings. Pain.

[126] Kuritzky A. (1984). Cluster headache-like pain caused by an upper cervical meningioma. Cephalalgia.

[127] Sharma R.R., Parekh H.C., Prabhu S., Gurusinghe N.T., Bertolis G. (1993). Compression of the C-2 root by a rare anomalous ectatic vertebral artery. J.Neurosurg..

[128] Hildebrandt J., Jansen J. (1984). Vascular compression of the C2 and C3 roots—Yet another cause of chronic intermittent hemicrania?. Cephalalgia.

[129] Hanzelka T., Dušek J., Ocásek F., Kučera J., Šedý J., Beneš J., Pavlíková G., Foltán R. (2013). Movement of the patient and the cone beam computed tomography scanner: Objectives and possible solutions. Oral Surg. Oral Med. Oral Pathol. Oral Radiol..

[130] Minervini G. (2022). Teledentistry in the management of patients with dental and temporomandibular disorders. Biomed Res. Int..

[131] Haas M., Bronfort G., Evans R., Schulz C., Vavrek D., Takaki L., Hanson L., Leininger B., Neradilek M.B. (2018). Dose-response and efficacy of spinal manipulation for care of cervicogenic headache: A dual-center randomized controlled trial. Spine J..

[132] Jull G., Trott P., Potter H., Zito G., Niere K., Shirley D., Emberson J., Marschner I., Richardson C. (2002). A randomized controlled trial of exercise and manipulative therapy for cervicogenic headache. Spine.

[133] Thompson J.R., Brody A.G. (1942). Factors in the position of the mandible. J. Am. Dent. Assoc..

[134] Hansson T., Henée W., Hesse J. (1990). Funktionsstörungen im Kausystem.

[135] Hansson T.L., Christensen Minor C.A., Wagnon Taylor D.L. (1992). Physical Therapy in Craniomandibular Disorders.

[136] Freesmeyer W.B. (1993). Zahnärztliche Funktionstherapie.

[137] Gelb H. (1994). New Concepts in Craniomandibular and Chronic Pain Management.

[138] Bergbreiter C. (1993). Untersuchung über die Zusammenhänge Zwischen der Fehlstatik und den Funktionellen Befunden des Craniomandibulären Systems.

[139] Stute W., Becker W. (1996). Sakrokraniomandibuläre Integrationsstörungen. Ganzheitliche Zahnheilkunde in der Praxis.

[140] Wallace C., Klineberg I. (1994). Management of Craniomandibular Disorders. Part II: Assessment of Patients with Craniocervical Dysfunction. J. Orofacial Pain.

[141] Coy R.E., Flocken J.E., Adib F. (1991). Musculoskeletal etiology and therapy of craniomandibular pain and dysfunction. Cranio Clin. Int..

[142] Shup W., Zernial P. (1996). Zahnärztliche und kieferorthopädische Behandlungsmöglichkeiten bei Craniomandibulärer Dysfunktion. Fachvereinigung deutscher Kieferorthopäden. (KFO-1G).

[143] Rocabado M. (2021). Atlas Clínico II, Congruencia Cráneo-cérvico-mandibular, Aplicación Clínica.

[144] Aniri M., Jull G., Bullock-Saxton J., Darnell R., Lander C. (2007). Cervical musculoskeletal impairment in frequent intermittent headache. Part 2: Subjects with concurrent headache types. Cephalgia.

[145] Dreyfuss P., Dreser S.J., Cole A., Mayo K. (2004). Sacroiliac joint pain. J. Am. Acad. Orthop. Surg..

[146] Hilton J. (1863). On Rest and Pain: A Course of Lectures on the Influence of Mechanical and Physiological Rest in the Treatment of Accidents and Surgical Diseases, and the Diagnostic Value of Pain, delivered at the Royal College of Surgeons of England in the years 1860, 1861, and 1862.

[147] Rocabado M., Gutierrez R., Gutierrez M.F., Gutierrez M.J. (2021). Case report: Anterior open bite correction treatment by dental treatment and physical therapy through craniocervical mandibular and occlusal stabilization. Cranio.

[148] Kang J.H. (2021). Neck associated factors related to migraine in adolescents with painful temporomandibular disorders. Acta Odontol Scand..

[149] von Piekartz H., Lüdtke K. (2011). Effect of treatment of temporomandibular disorders (TMD) in patients with cervicogenic headache: A single-blind, randomized controlled study. Cranio.

[150] Williamson E.H. (1981). Eugene, H. Williamson on occlusion and TMJ dysfunction. Interview by S. Brandt. J. Clin. Orthod.

[151] Williamson E.H. (1981). Eugene, H. Williamson on occlusion and TMJ dysfunction (Part 2). J. Clin. Orthod..

[152] Dawson P.E. (2007). Functional Occlusion: From TMJ to Smile Design.

[153] Greven G., Piehslinger E., Haberl T., Betzl C. (2020). Correlation between Internal Derangement of the Temporo-Mandibular Joint and Ipsi-Lateral Mediotrusive Molar Interferences-A Condylographic Study Using Virtual Articulation. Int. J. Dent. Oral Health.

[154] Gross A., Langevin P., Burnie S.J., Bédard-Brochu M.S., Empey B., Dugas E., Faber-Dobrescu M., Andres C., Graham N., Goldsmith C.H. (2015). Manipulation and mobilisation for neck pain contrasted against an inactive control or another active treatment. Cochrane Database Syst. Rev..

[155] Chen L., Zhang X.L., Ding H., Tao Y.Q., Zhan H.S. (2007). Comparative study on effects of manipulation treatment and transcutaneous electrical nerve stimulation on patients with cervicogenic headache. J. Chin. Integr. Med./Zhong. Xi. Yi..

[156] Deyo R.A., Walsh N.E., Schoenfeld L.S., Ramamurthy S. (1990). Can trials of physical treatments be blinded? The example of transcutaneous electrical nerve stimulation for chronic pain. Am. J. Phys. Med. Rehabil..

